# The Carbon Assimilation Network in *Escherichia coli* Is Densely Connected and Largely Sign-Determined by Directions of Metabolic Fluxes

**DOI:** 10.1371/journal.pcbi.1000812

**Published:** 2010-06-10

**Authors:** Valentina Baldazzi, Delphine Ropers, Yves Markowicz, Daniel Kahn, Johannes Geiselmann, Hidde de Jong

**Affiliations:** 1 Institut National de Recherche en Informatique et en Automatique, INRIA Grenoble - Rhône-Alpes, Montbonnot, France; 2 Laboratoire Adaptation et Pathogénie des Microorganismes (CNRS UMR 5163), Université Joseph Fourier, Bâtiment Jean Roget, Faculté de Médecine-Pharmacie, La Tronche, France; 3 Laboratoire de Biométrie et Biologie Evolutive (CNRS UMR 5558), Université Lyon 1, INRA, Villeurbanne, France; Harvard University, United States of America

## Abstract

Gene regulatory networks consist of direct interactions but also include indirect interactions mediated by metabolites and signaling molecules. We describe how these indirect interactions can be derived from a model of the underlying biochemical reaction network, using weak time-scale assumptions in combination with sensitivity criteria from metabolic control analysis. We apply this approach to a model of the carbon assimilation network in *Escherichia coli*. Our results show that the derived gene regulatory network is densely connected, contrary to what is usually assumed. Moreover, the network is largely sign-determined, meaning that the signs of the indirect interactions are fixed by the flux directions of biochemical reactions, independently of specific parameter values and rate laws. An inversion of the fluxes following a change in growth conditions may affect the signs of the indirect interactions though. This leads to a feedback structure that is at the same time robust to changes in the kinetic properties of enzymes and that has the flexibility to accommodate radical changes in the environment.

## Author Summary

The regulation of gene expression is tightly interwoven with metabolism and signal transduction. A realistic view of gene regulatory networks should therefore not only include direct interactions resulting from transcription regulation, but also indirect regulatory interactions mediated by metabolic effectors and signaling molecules. Ignoring these indirect interactions during the analysis of the network dynamics may lead crucial feedback loops to be missed. We present a method for systematically deriving indirect interactions from a model of the underlying biochemical reaction network, using weak time-scale assumptions in combination with sensitivity criteria from metabolic control analysis. This approach leads to novel insights as exemplified here on the carbon assimilation network of *E. coli*. We show that the derived gene regulatory network is densely connected, that the signs of the indirect interactions are largely fixed by the direction of metabolic fluxes, and that a change in flux direction may invert the sign of indirect interactions. Therefore the feedback structure of the network is much more complex than usually assumed; it appears robust to changes in the kinetic properties of its components and it can be flexibly rewired when the environment changes.

## Introduction

The adaptation of bacteria to changes in their environment involves adjustments in the expression of genes coding for enzymes, regulators, membrane transporters, *etc.* [1]–[3]. These adjustments are controlled by gene regulatory networks ensuring the coordinated expression of clusters of functionally related genes. The interactions in the network may be direct, as in the case of a gene coding for a transcription factor regulating the expression of another gene. Most of the time, however, regulatory interactions are indirect, *e.g.* when a gene encodes an enzyme producing a transcriptional effector [4].

A gene regulatory network can thus not be reduced to its transcriptional regulatory interactions: by ignoring indirect interactions mediated by metabolic and signaling pathways we may miss crucial feedback loops in the system. The network controlling carbon uptake in the bacterium *Escherichia coli* is a good example because it integrates metabolism, signal transduction, and gene expression. At the level of gene expression, the network includes intricate feedback loops that arise from indirect interactions between the subsystems. Global regulators like Crp control expression of enzymes in carbon metabolism [5]–[8], while intermediates of the latter pathways control the expression of global regulators. For instance, the phosphorylation of EIIA activates adenylate cyclase (Cya) to produce cAMP which is required for the activation of Crp [9], [10].

The aim of this paper is to develop a method for the systematic derivation of direct and indirect interactions in a gene regulatory network from the underlying biochemical reaction network. Due to the complexity of the intermediate metabolic and signaling networks, determining indirect interactions is difficult in general. We show that model reduction based on quasi-steady-state (QSS) approximations expressing weak assumptions on time-scale hierarchies in the system [11]–[13], together with sensitivity criteria from metabolic control analysis (MCA) [12], [14], are able to uncover such interactions. Indeed the MCA formalism uniquely allows to relate systemic sensitivities (‘control coefficients’) with the sensitivities of individual reactions to reactants and effectors [12], [15]. It therefore provides a proper framework for investigating metabolic effects in gene regulation.

We apply our approach to a model of the upper part of the carbon assimilation network in *E. coli*, consisting of the glycolysis and gluconeogenesis pathways and their genetic and metabolic regulation. The analysis of the derived gene regulatory network leads to three new insights. First, contrary to what is often assumed, the network is densely connected due to numerous feedback loops resulting from indirect interactions. This additional complexity is an important issue for the correct interpretation of data from genome-wide transcriptome studies. Second, the derived gene regulatory network for carbon assimilation in *E. coli* is sign-determined, in the sense that the signs of interactions are essentially fixed by weak information on flux directions of biochemical reactions, without explicit specification of kinetic rate laws or parameter values. Therefore the feedback structure is robust to changes in kinetic properties of enzymes and other biochemical reactions species. Third, a change in environmental conditions may invert fluxes, and thus the signs of indirect interactions, resulting in a dynamic rewiring of the regulatory network.

## Methods

### Model reduction

We used standard approaches from biochemistry to build a kinetic model of the network of glucose assimilation in *E. coli*. The model describes the genetic and metabolic regulation of glycolysis and gluconeogenesis. The model takes the form of a system of ordinary differential equations (ODEs), describing the rate of change of the concentrations of proteins, RNAs and metabolites:
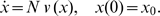
(1)

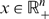
 denotes the vector of concentrations and 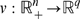 the vector of reaction rates. 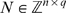 is a stoichiometry matrix. In the presence of conserved quantities, 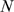 is reformulated in such a way that the dependencies between variables are eliminated [16]. In the following, we assume that 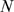 is such a reduced matrix.

Eq. 1 can be simplified by applying the QSS approximation [12]. Two different time-scales are distinguished, one corresponding to the slow processes (protein synthesis and degradation) and one to the fast processes (complex formation and enzymatic reactions). Considering metabolic processes as fast is justified when metabolic pools undergo turnover times in the range of seconds, as is the case for the very active glycolysis in *E. coli* [17]. Therefore, we introduce vectors of slow and fast variables, 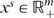 and 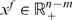, respectively (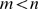), defined as linear combinations of the original variables 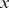:
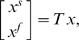
(2)with 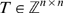. The slow variables typically correspond to total protein concentrations, whereas the fast variables include concentrations of metabolites and biochemical complexes:
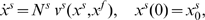
(3)

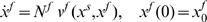
(4)where 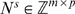 and 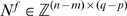 are stoichiometry matrices for the slow and fast part, respectively, and 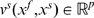 and 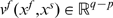 the corresponding reaction rates (see Sec. 1 of Supporting Information Text S1 for details).

The QSS hypothesis states that at the time-scale of the slow processes, the fast part of the system can be assumed to be at steady state, instantly adapting to the dynamics of the slow variables, *i.e.* 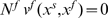. The conditions for the applicability of this approximation are given by the Tikhonov theorem, which imposes exponential stability of the fast system [12]. The stability of metabolism in its normal range of operation is a reasonable assumption in most situations.

### Derivation of interaction structure

The QSS approximation implicitly relates the steady-state values of the fast variables to the concentrations of the slow variables, *i.e.* 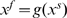, 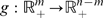, if such a function can be found. The resulting system at the slow time-scale has the following form
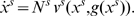
(5)This reduced model makes explicit the fact that the biochemical reactions in the fast subsystem induce additional interactions between the slow variables. For metabolic systems the QSS equation is nonlinear in terms of 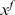 and it is generally impossible to obtain a closed-form expression for the function 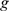. We therefore follow another strategy to characterize the indirect interactions between the slow variables, that is, the regulation of gene expression via metabolic intermediates. We study the Jacobian matrix 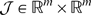 of the system in Eq. 5, which captures the interaction structure of the gene regulatory network:
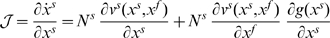
(6)The Jacobian matrix includes the direct effect of each slow variable on the others (first term) and the indirect effect via the coupling through the fast system (second term). It accounts for direct regulation of gene expression by transcription factors as well as indirect regulation through metabolism. The indirect regulation involves both the effect of changes in fast variables on the rates of slow variables (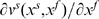) and the effect of changes in slow variables on QSS values of fast variables (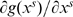). The former effect can be directly determined from the rate equations, as it describes, for instance, the regulation of a gene by a metabolic effector. The latter effect expresses the sensitivity of the metabolic state to changes in the slow variables, which corresponds to concentration control coefficients in the framework of MCA [12], [16].

Implicit differentiation of the QSS equation 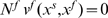 results in
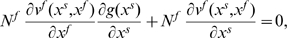
(7)which describes the response of the fast system around its steady state to changes in the slow variables. Notice that 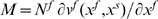 corresponds to the Jacobian matrix of the fast system. The reduction of 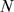 for conserved quantities assures that 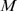 is not singular (see Sec. 1 of Text S1). Therefore, if the steady state is stable then, using Eq. 7 and the definition of 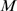, concentration control can be expressed [16] as
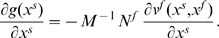
(8)The latter formula can then be substituted into Eq. 6, the expression of the Jacobian matrix of the slow system. 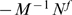 is the matrix of non-normalized concentration control coefficients [12], [16].

The computation of 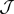 as described above requires the manipulation of complex algebraic expressions. As this is too cumbersome and error-prone to do by hand, the process has been implemented by means of the Symbolic Math Toolbox of MATLAB (MathWorks). Inversion of large symbolic matrices like 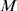 is a computationally challenging task, but the matrices considered in the *E. coli* example are within the reach of state-of-the-art computer algebra tools. The computations take a few seconds to complete on a PC (Intel Core 2, 1.86 GhZ, 2 Gb of RAM).

### Determination of signs of interactions

The rate vectors 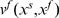 and 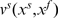 are typically nonlinear functions involving many parameters with unknown values. However, since 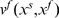 and 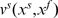 are usually monotonic functions of the variables, the signs of the partial derivatives in Eqs. 6 and 8 are fixed over the entire state space. This information can be used to evaluate the sign of the elements of 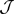.

**Figure 1:**
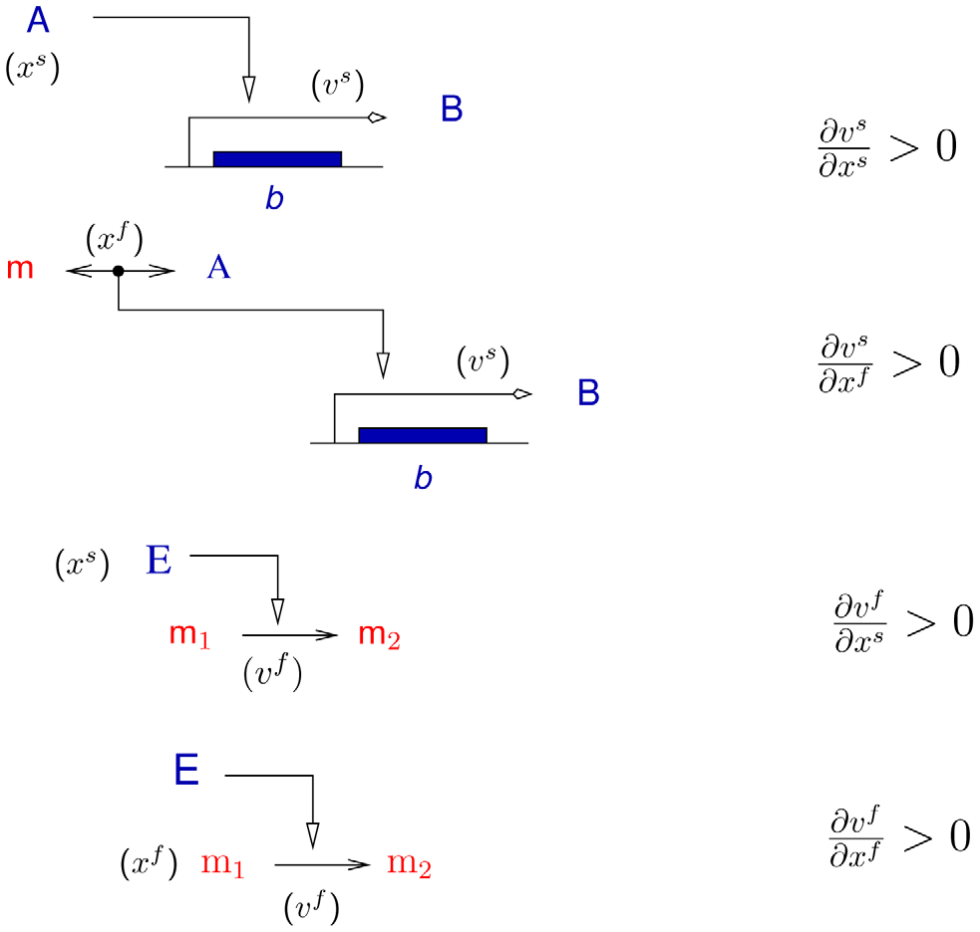
Schematic representation of the partial derivatives of the rates vectors 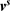 and 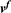, appearing in Eqs. 6 and 8. An example is provided for each of the four partial derivatives, and its corresponding sign is indicated on the right side. A and B are proteins, E an enzyme, and m, m_1_, and m_2_ metabolites.

This argument can be clarified by considering the partial derivatives of the rates occurring in Eqs. 6 and 8 (see Fig. 1 for a schematic illustration). 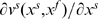 describes the direct interactions between slow variables, typically the control of gene expression by a transcriptional regulator. The signs of these interactions are in general unambiguously given by the literature [5]. We omit the special case of non-specific degradation and growth dilution, which are not usually interpreted as regulatory interactions [18]. 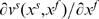 describes the direct relations between the fast and slow parts of the system through fast coupling species, *e.g.*, a transcriptional regulator whose activity is modified by a metabolite: their signs are known. 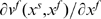 accounts for the direct influence of fast variables on the fast dynamics, typically the variation of enzyme rates with a change in concentration of substrate, product, or effector. Given a convention on the positive flux direction, the signs of these elasticities are usually unambiguously defined, except in rare cases of substrate inhibition or product activation. In such a case our analysis pertains provided the ranges of concentrations are restricted so that enzyme rates remain monotonic functions of concentrations. Finally, 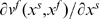 describes the direct influence of slow variables on the fast dynamics, typically the variation of a reaction rate with a change in enzyme concentration. In this case 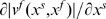 is positive because absolute values of reaction rates increase with enzyme concentration, so that the sign of this effect is solely determined by the direction of the flux (equal to its sign). Therefore a change in growth conditions implies a switch of the signs of some interactions, whenever there is a change in flux direction. For instance in the carbon assimilation model, different regulatory patterns will emerge depending on whether the bacteria grow on glycolytic or gluconeogenic substrates.

When do the signs of the partial derivatives of the rates unambiguously fix the signs of the structure of interactions between the slow variables? Analysis of the Jacobian matrix in Eq. 6 reveals that the following four conditions are sufficient to obtain what we call a sign-determined network (see Sec. 2 of Text S1).

**(C1)** A slow variable acts directly either on the slow system or on the fast system, but not on both simultaneously. In practice this excludes enzymes as transcriptional regulators or moiety conserved species as transcriptional effectors. Under this condition at most one of the terms in Eq. 6 is non-zero for each element of 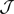.

**(C2)** No variable has direct antagonistic (*i.e.*, both activating and inhibiting) effects on a slow variable. This means, for example, that a transcription factor cannot both activate and inhibit the expression of the same gene (no mixed regulation), although it may activate one gene and inhibit another.

**(C3)** The concentration control coefficients of the fast coupling species with respect to the slow variables have a determinate sign.

**(C4)** If a slow variable contributes to the concentration control of several fast coupling species, the latter do not simultaneously regulate any of the slow variables (no concerted regulation). Together **C3** and **C4** guarantee that the second term in the right-hand side of Eq. 6 is unequivocally defined.

**Figure 2:**
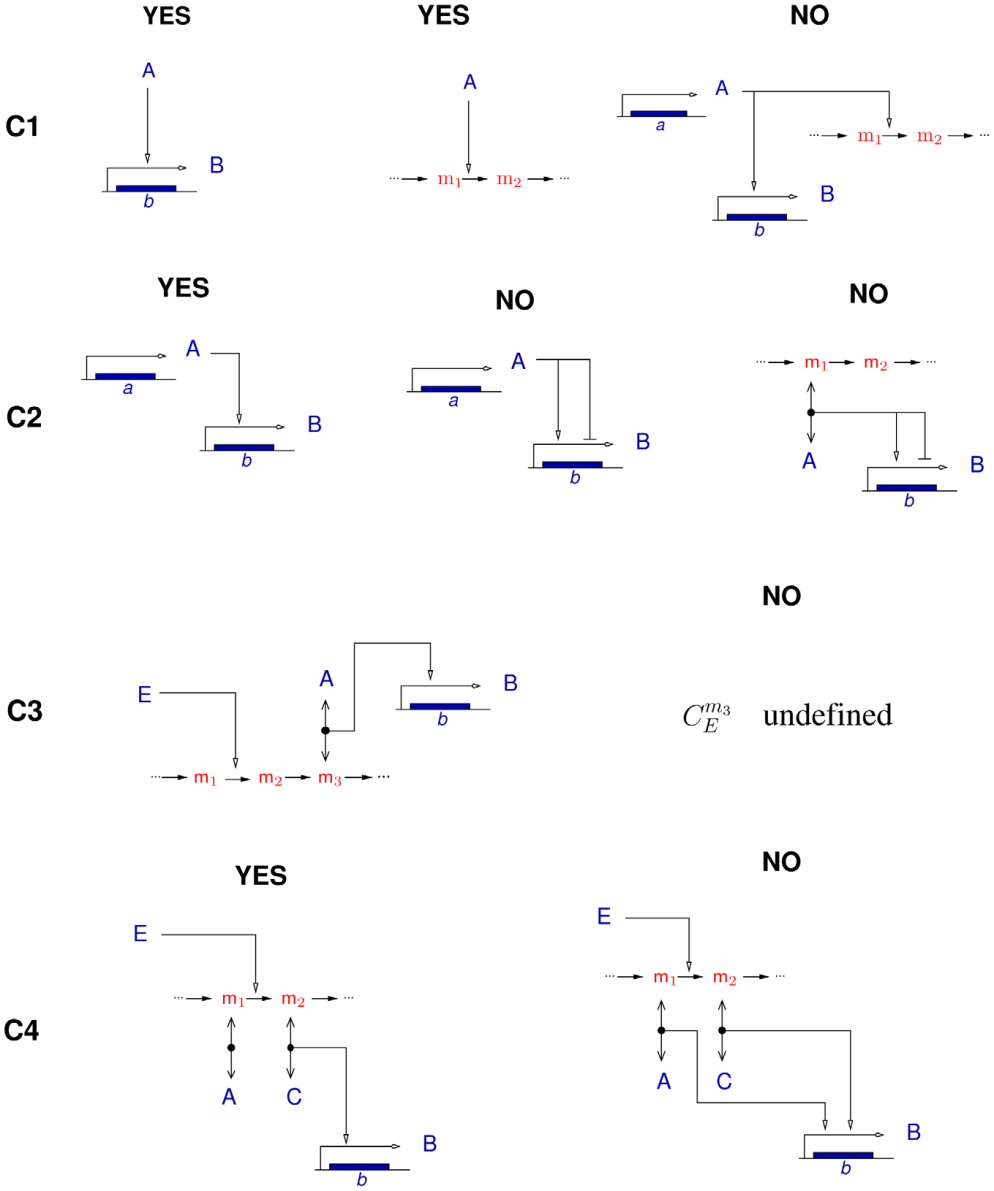
Schematic illustration of the four sufficient conditions for sign determinedness, C1–C4. The conditions are explained by means of example patterns that are either allowed or forbidden. Notice that the above examples are not meant to be exhaustive; their aim is simply to clarify the consequences of **C1**–**C4** on a few concrete cases. In the examples, A, B, C, and E are proteins, while m_1_, m_2_, and m_3_ are metabolites. 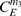 is the concentration control coefficient of m_3_ with respect to the reaction catalyzed by E.
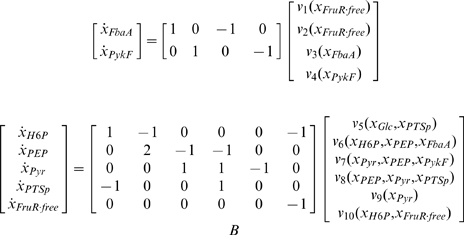

Fig. 2 illustrates the four conditions **C1**–**C4** in terms of allowed and forbidden patterns in the biochemical reaction system.

Notice that these conditions do not give the actual signs of the elements of 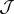, but they help in relating the sign-(un)determinedness of the network to specific features of the underlying biochemical reaction system. Whereas **C1** and **C2** are not very restrictive, the satisfaction of especially **C3** is not evident in practice. In the case of a metabolic network with a complex structure, involving substrate cycles or allosteric regulation, antagonistic effects may compete in the control of concentration. Such situations were analyzed previously in the framework of MCA [19]. For instance, the signs of concentration control coefficients are frequently undetermined for metabolites on the path between an allosteric effector and its target. Another case of undeterminedness concerns substrate cycles. Whenever such antagonistic effects arise, additional information will be required on the relative magnitudes of opposing effects.

The stability underlying the QSS approximation imposes additional constraints that can be exploited to resolve ambiguities. A classical result in linear system theory [20] states a necessary condition for the stability of the fast system, namely that the coefficients of the characteristic polynomial
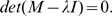
(9)all have the same sign. This provides an independent set of inequalities between partial derivatives that can be used to estimate the signs of control coefficients in Eq. 8 and thus satisfy **C3**.

**Figure 3:**
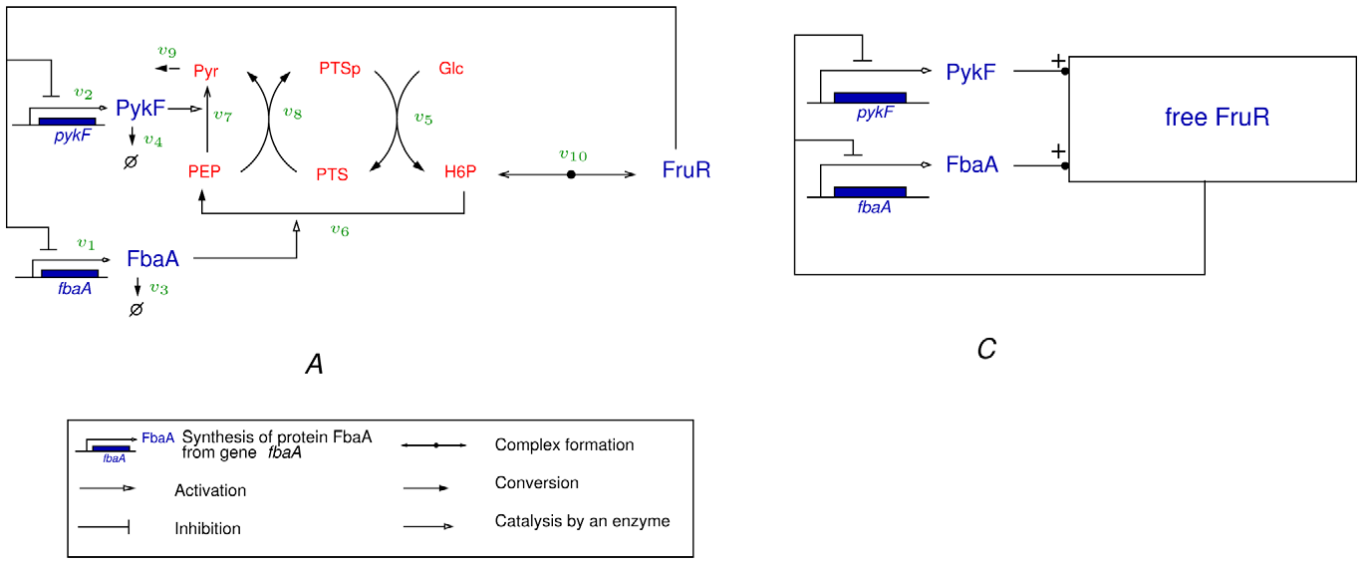
Simplified glycolysis model, adapted from [**30**], including the genetic regulation of enzyme expression by FruR. *A*: Network of biochemical reactions. The conversion of a generic hexose-6-phosphate H6P to PEP is schematized as a single reaction, with FbaA being assumed representative of all glycolytic enzymes. The network also includes a simplified description of the PTS, including the phosphorylated and non-phosphorylated form of its enzymes (represented by PTSp and PTS, respectively). The total concentration of the PTS enzymes is assumed constant. FruR is inactivated when bound to fructose-6-phosphate, here represented by H6P. The total (bound and unbound) FruR concentration is assumed constant in this example. The reactions correspond to protein synthesis (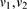), protein degradation (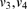), enzymatic reactions (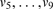), and complex formation (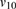). Proteins are shown in red, metabolites in blue, and reactions in green. *B*: Kinetic model of the network in the form of Eq. 3 and 4, separating the slow (protein synthesis and degradation) from the fast processes (enzymatic reactions and complex formation). The corresponding slow variables are the total enzyme concentrations (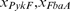), while the fast variables are the metabolite concentrations (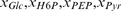), the concentration of FruR protein in its free form (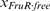), and the phosphorylated PTS enzyme (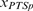). *C*: Complete network of direct and indirect gene regulatory interactions, with unequivocal signs for the influences of the enzymes on the concentration of free FruR. The interactions are derived from the Jacobian matrix of the slow system (for the detailed analysis of the network, see Text S2).

Fig. 3 shows the network of direct and indirect gene regulatory interactions computed for a simplified model of the carbon assimilation network. The model describes the main reactions involved in the control of the glycolysis pathway, during growth on glucose (Fig. 3*A*). In particular it accounts for the genetic regulation of enzymes levels, and thus provides an interesting example for the analysis of indirect interactions arising from the coupling between gene expression and metabolism. The corresponding ODE system, written in the form (3)–(4), is shown in Fig. 3*B*. Application of the method explained above results in the appearance of novel interactions between genes *fbaA* and *pykF*, mediated by the fast coupling species free FruR (see Fig. 3*C*). These interactions are not expected on the basis of a purely transcriptional control. The derivation of the interactions from the model are described in detail in Text S2. In this case, the stability condition is sufficient to satisfy all conditions and make the network sign-determined.

## Results

### Model of carbon assimilation network

**Figure 4:**
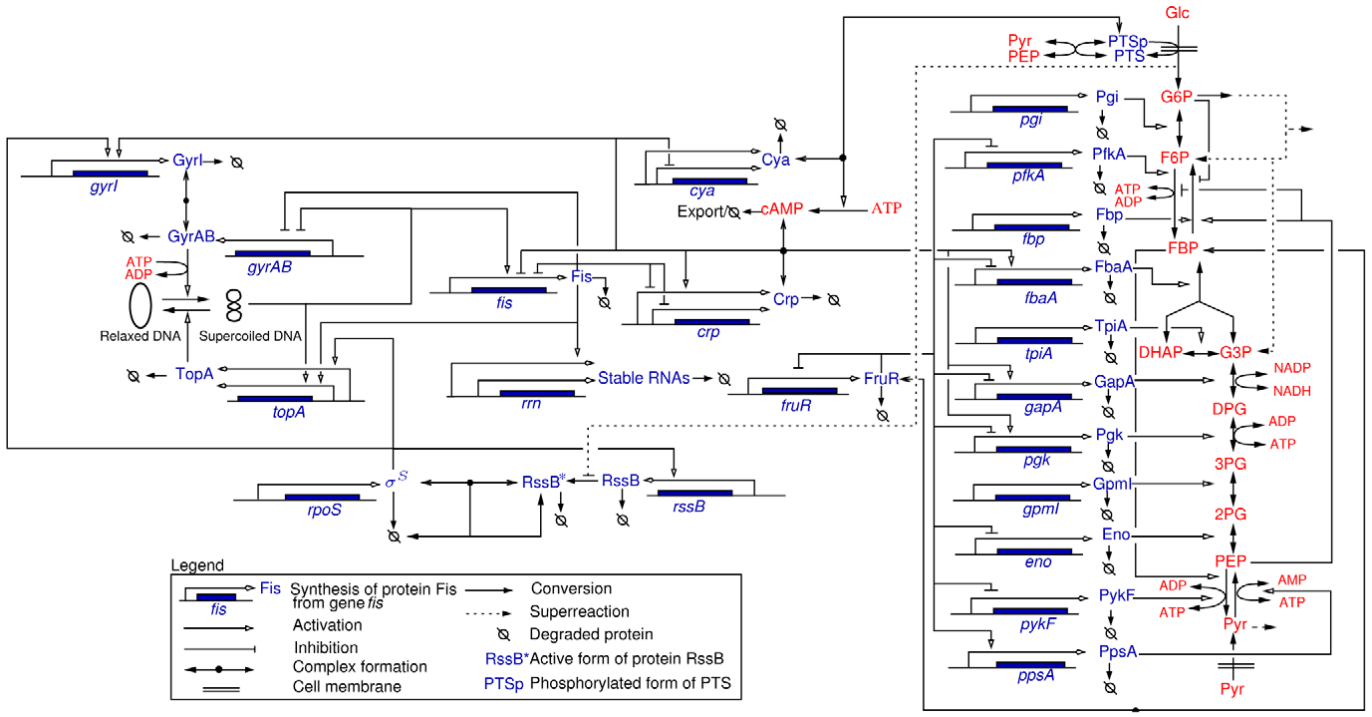
Network of key genes, proteins, and regulatory interactions involved in the carbon assimilation network in *E. coli*. The graphical conventions [52] are explained in the legend. The metabolic part includes the glycolysis/gluconeogenesis pathways as well as a simplified description of the PTS system (adapted from [30]), via the phosphorylated and non-phosphorylated form of its enzymes (represented by PTSp and PTS, respectively). The pentose-phosphate pathway (PPP) is not explicitly described but we take into account that a small pool of G6P escapes the upper part of glycolysis. Graphically, the PPP is represented as a ‘super-reaction’, in which elementary steps are lumped together. At the level of the global regulators the network includes the control of the DNA supercoiling level, the accumulation of the sigma factor RpoS and the Crp

Glucose is the preferred carbon source of *E. coli* and its assimilation is tightly regulated in the cell. This control involves a signaling pathway and transport system (PTS), a modification of metabolic activities (glycolysis, TCA cycle, pentose-phosphate pathway, gluconeogenesis), and the regulation of gene expression (glycolytic and gluconeogenic enzymes, global regulators). These different modes of control have mostly been studied in isolation, whereas in fact they are interwoven and form a large and complex regulatory network. In this study we focus on the part of the regulatory network controlling glycolysis and gluconeogenesis. Briefly, this network accounts for the sensing and uptake of glucose via the PTS, its conversion to pyruvate, as well as the regeneration of more complex sugars from pyruvate when the latter is used as a carbon source (Fig. 4). At the level of gene expression we consider genes coding for metabolic enzymes and their key regulators, *fis*, *crp*, and *fruR* [6], [8]. In addition, we include the general stress factor RpoS and the regulators of DNA topology (GyrAB, TopA, …), as changes in the superhelicity of DNA affect the expression of many of the above-mentioned genes [7].

cAMP complex, and the regulatory role exerted by the fructose repressor FruR. A complete description of the model can be found in Sec. 1 of Text S3. Proteins are shown in red, and metabolites in blue.

Changes in gene expression modify the concentrations of enzymes, and thus of intracellular fluxes and metabolite concentrations. A critical point in the regulation of carbon assimilation is the pair of reactions interconverting PEP and Pyr, involving the differentially regulated enzymes PykF and PpsA, required respectively for glycolysis and gluconeogenesis [21], [22]. Metabolism also acts back on gene expression. For instance, FBP and cAMP are two key metabolites that modulate the activity of the transcription regulators FruR and Crp, respectively [6], [10], [23], [24]. The PTS plays a special role in this context by converting information on glucose availability into an activation signal for cAMP synthesis, thus inducing a reorganization of global gene expression by Crp

cAMP [10], [25]–[27].

We have developed a model that describes the coupling between metabolism and gene expression, consisting of 66 reactions and involving 40 species. The model is based on existing models of carbon metabolism [27], [28] and global regulators of gene expression [29], which include the experimentally validated interactions reported in the literature (see Text S3). However, contrary to these models, we do not specify kinetic rate laws, as only the signs of the partial derivatives are used for reconstructing the (signs of) indirect interactions. We apply the QSS approximation by distinguishing two distinct time-scales in the system: a fast time-scale for complex formation, DNA supercoiling and all reactions involved in glycolysis, gluconeogenesis, PTS signaling, and cAMP production, and a slow time-scale for the synthesis and degradation of global regulators, enzymes and stable RNAs. The equations of the original and the reduced model, as well as the different approximation steps, are described in detail in Secs.1 and 2 of Text S3.

For analytical purposes, four variants of the model are analyzed below, accounting for differences in growth conditions and regulatory effects. The differences concern only a few of the 66 reactions. We consider two possible carbon sources, glucose or pyruvate, thus imposing a fixed direction on reactions. Some reactions have negligible flux, such as the PEP synthase during glycolysis [21]. Glycolysis and gluconeogenesis are therefore treated separately by two distinct models 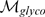 and 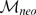. For each of these we define two variants that do not or do include allosteric regulation of enzyme activities: 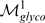, 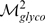, respectively, for glycolysis and 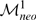, 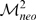, respectively, for gluconeogenesis.

### Sign-determinedness of gene regulatory network

The coupling between metabolism and gene regulation leads to additional, indirect dependencies between genes. We first focus on the networks obtained in the absence of allosteric regulation, using models 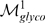 and 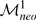. Application of the method introduced above to the glycolytic model, as described in Sec. 3 of Text S3, results in the sign pattern of the Jacobian matrix in Table 1.

**Table 1:** Interaction matrix of the gene regulatory network for the glycolytic mode.

	PfkA	FbaA	GapA	Pgk	Eno	PykF	Cya	Crp	Fis	GyrAB	GyrI	TopA	RpoS	RssB	stable RNAs	FruR
*pfkA*	0	**−**	**−**	**−**	**−**	**−**	0	0	0	0	0	0	0	0	0	**−**
*fbaA*	0	**−**(**−/+**)	**−**(**−/+**)	**−**(**−/+**)	**−**(**−/+**)	**−**	**+**	**+**	0	0	0	0	0	0	0	**−**
*gapA*	0	**−**(**−/+**)	**−**(**−/+**)	**−**(**−/+**)	**−**(**−/+**)	**−**	**+**	**+**	0	0	0	0	0	0	0	**−**
*pgk*	0	**−**(**−/+**)	**−**(**−/+**)	**−**(**−/+**)	**−**(**−/+**)	**−**	**+**	**+**	0	0	0	0	0	0	0	**−**
*eno*	0	**−**	**−**	**−**	**−**	**−**	0	0	0	0	0	0	0	0	0	**−**
*pykF*	0	**−**	**−**	**−**	**−**	**−**	0	0	0	0	0	0	0	0	0	**−**
*cya*	0	0 (**−**)	0 (**−**)	0 (**−**)	0 (**−**)	**+**	**−**	**−**	0	0	0	0	0	0	0	0
*crp*	0	0 (**+**)	0 (**+**)	0 (**+**)	0 (**+**)	**−**	**+**	**+**	**−**	0	0	0	0	0	0	0
*fis*	0	0	0	0	0	0	**−**	**−**	**−**	**+**	**−**	**−**	0	0	0	0
*gyrAB*	0	0	0	0	0	0	0	0	**−**	**−**	**+**	**+**	0	0	0	0
*gyrI*	0	0	0	0	0	0	**+**	**+**	0	0	0	0	**+**	0	0	0
*topA*	0	0	0	0	0	0	0	0	**+**	**+**	**−**	**−**	**+**	0	0	0
*rpoS*	0	0	0	0	0	0	0	0	0	0	0	0	0	**−**	0	0
*rssB*	0	0	0	0	0	0	0	0	0	0	0	0	**+**	0	0	0
*rrn*	0	0	0	0	0	0	0	0	**+**	0	0	0	0	0	0	0
*fruR*	0	**−**	**−**	**−**	**−**	**−**	0	0	0	0	0	0	0	0	0	**−**

The matrix describes the effect of regulators (column) on genes (rows). Plus signs stand for activation of a gene by a regulator, and minus signs for inhibition. In determining the signs, we excluded the direct effect of a slow variable on itself when the latter is due to non-specific protein degradation through decay and growth dilution [18]. Signs in brackets correspond to interactions whose signs are different in the case of allosteric regulation (that is, they are changed when using model 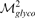 instead of model 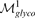). The double sign for the effect of enzymes FbaA, GapA and Pgk on genes *fbaA*, *gapA* and *pgk* describes the combined control of free FruR and Crp

cAMP in the presence of allosteric regulation. In particular, the regulation exerted by free FruR leads to an inhibition, whereas a positive regulation arises from allosteric effects via Crp

cAMP.

Several novel indirect interactions appear, some of which are straightforward, like the inhibitory effect of Crp on *cya* through Crp

cAMP. Others, however, are less evident or even counter-intuitive such as the predicted negative control of the expression of the global regulator FruR by enolase (Eno) during growth on glucose. This effect is explained by the fact that an increase in *eno* expression leads to a reduced FBP concentration, and thus to an increased *fruR* downregulation.

The most striking result of our analysis is that the signs of the indirect interactions are uniquely defined, that is, during growth on glucose, the proteins exert an unambiguous effect (zero, positive or negative) on their target genes. The signs of these indirect interactions are therefore a structural property of the underlying system of biochemical reactions. The same result is observed in the case of growth on pyruvate, for the gluconeogenic model 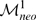 (Sec. 3 of Text S3). The sign-determinedness of the network can be analyzed by means of the conditions **C1**–**C4**. 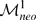 satisfies all sufficient conditions for sign-determinedness. In particular, the concentration control coefficients acting on coupling species have a unique sign, as requested by **C3**. 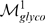 satisfies **C1**–**C3**, but not **C4**. The concerted regulation excluded by **C4** does not pose a problem for sign-determinedness in this particular case, however, because PykF has the same effect through both fast coupling species Crp

cAMP and free FruR.

### Allosteric regulation and sign-determinedness

Allosteric regulation is important for metabolism, but adds a level of complexity that may affect the sign-determinedness of the network. We verified this by applying the method to the glycolytic model with allosteric regulation, 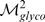. The latter model notably includes the positive regulation of PykF activity by FBP [30], [31] and the inhibitory effect of PEP on PfkA [27].

As a consequence of the feedforward loop from FBP to PykF, **C3** and **C4** do no longer hold for 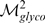, and in fact the network becomes partially sign-undetermined. In particular, the glycolytic enzymes FbaA, GapA, Pgk, and Eno exert antagonistic effects on the control of the concentration of free FruR, thus invalidating **C3**. Moreover, the presence of allosteric regulation results in a denser Jacobian matrix 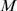 of the fast system. This causes some of the glycolytic enzymes to contribute to the control of both Crp

cAMP and free FruR. Contrary to **C4**, these fast coupling species simultaneously regulate the genes coding for three of the enzymes, in antagonistic ways.

By means of conditions **C1**–**C4** the partial sign-undeterminedness can thus be related to specific network features. Interestingly, it also enables one to identify experiments that would resolve sign ambiguities: indeed, a single observation, measuring the response of the FBP concentration to an increased expression of FbaA, would allow us to unequivocally determine the signs of all control coefficients (Sec. 3 of Text S3). Such an observation has been reported in the literature [32] and makes condition **C3** true. The resulting signs of the control coefficients are the same as for the model without allosteric effects, thus indicating that the regulation of PykF activity by FBP finetunes rather than inverses the concentration control of the system.

**Figure 5:**
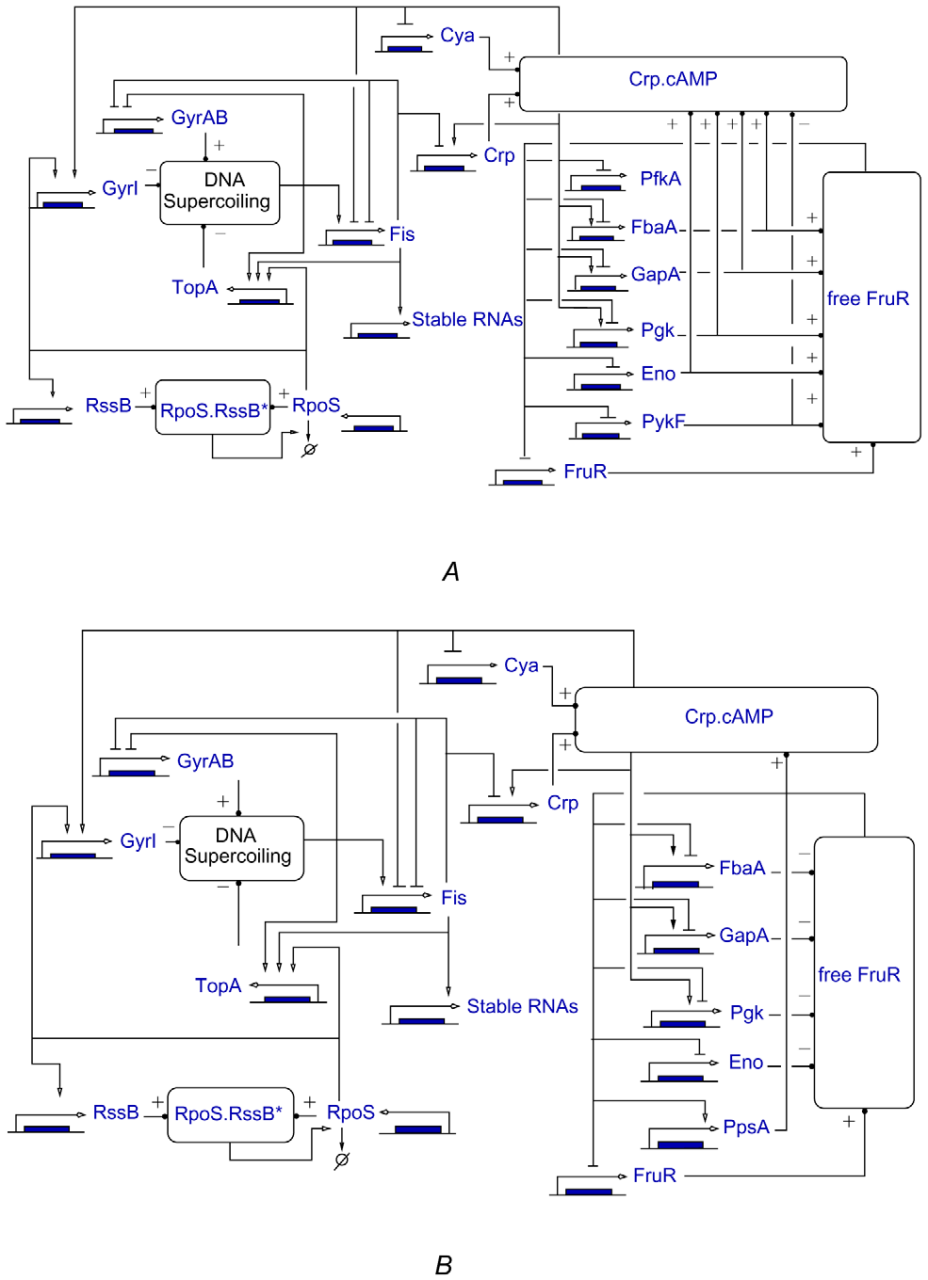
Networks of direct and indirect regulatory interactions in the case of (*A*) glycolysis and (*B*) gluconeogenesis. The networks are computed from the models 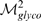 and 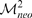, taking into account allosteric effects (see Sec. 3 of Text S3). The boxes represent fast coupling species, mediating the influence of metabolism and signal transduction on gene expression: Crp

The derived gene regulatory network during glycolysis, after disambiguation of the concentration control coefficients, is shown in Fig. 5*A*. The experimental data do not resolve the ambiguities invalidating **C4**. In particular, the regulation mediated by Crp

cAMP leads to an activation whereas free FruR is responsible for a negative control. In this situation, the resulting net effect of these regulators on their targets cannot be predicted without information on the parameters or gene expression patterns under glycolytic growth conditions, and a double sign appears in Table 1. Notice however that this concerns only 12 out of 256 entries in the Jacobian matrix 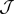 describing the interaction structure. The network is found to be completely sign-determined in gluconeogenesis, even when taking into account allosteric regulation (Sec. 3 of Text S3).

cAMP, free FruR, RpoS

RssB*, and DNA supercoiling. The influence of the enzymes and other slow species on the concentration of the fast coupling species are represented by +/− signs.

### Interaction signs and growth conditions

The above analysis is based on the assumption that the net flux direction is fixed, which means that the obtained network is growth-condition specific: some indirect interactions appear under one growth condition and are absent in the other (Fig. 5). Moreover, the same interaction may have an opposite sign in the two cases, for instance the effect of Eno on the concentration of free FruR. This context-dependency of the regulatory structure is due to the fact that the concentration control exerted by the glycolytic enzymes on free FruR and Crp

cAMP, the two main connections between carbon metabolism and gene regulation, changes sign depending on whether the bacteria grow on glucose or pyruvate. More generally, it can be shown with MCA that concentration control coefficients change sign upon flux inversion, resulting in an inversion of the corresponding gene interactions. This shows that the structure of regulatory interactions may be dynamically rewired by the environment, which potentially enhances the adaptive capacity of the system.

### Densely connected network

Classically, gene regulatory networks are considered to be sparsely connected, with only a few regulators per gene [33]–[36]. Most of these studies, however, have focused on direct transcriptional regulations, without considering the indirect interactions arising from the coupling between metabolism and gene expression. As these indirect interactions are operative on the time-scale of the slow variables, they can not be ignored when studying the dynamics of the gene regulatory network, for instance in the context of transcriptome studies.

In order to assess the effect of including indirect interactions in the *E. coli* network, we have counted the average connectivity per gene and the number and the length of the feedback loops in the system (Sec. 4 of Text S3). We compare the results with a baseline model 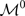 that only considers classical, direct interactions.

The carbon assimilation network of the baseline model 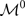 has an average connectivity of 1.4 regulatory proteins per gene. These values are in agreement with estimations made for *E. coli* and other organisms at the genomic scale [33]–[36]. Only four feedback loops are detected, most of which (3 out of 4 cases) are cases of direct autoregulation. The addition of indirect interactions changes the picture completely (Table 2). The average connectivity rises to over 4 and the number and length of feedback loops increases dramatically. Some feedback loops involve 12 elements, that is, 75% of the genes in the network.

**Table 2:** Structural features of the gene regulatory networks inferred from different models of the carbon assimilation network.

	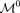	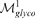	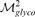	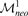	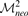
Number of feedback loops	4	2388	9246	24	2257
Maximal loop length	2	12	12	6	12
Average connectivity	1.4	4.7	5.2	2.8	4.4

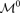
 corresponds to the transcriptional regulatory network in which indirect interactions mediated by metabolism are not taken into account.

The influence of metabolism on gene expression is channeled through a small number of intermediates, essentially Crp

cAMP and free FruR. Leaving out one of these coupling species immediately reduces the number and length of the feedback loops. For instance, eliminating the indirect interactions associated with Crp

cAMP reduces the number of feedback loops to a mere 20% of those present in Table 1, and the maximal loop length drops from 12 to 6. This agrees with the central role of Crp

cAMP in the control of carbon assimilation in *E. coli* [5], [6]. The effect of eliminating the interactions mediated by FruR is less dramatic, consistent with its more local role [5], [6], [37].

The comparison of the models with and without allosteric regulation (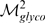 vs 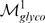, 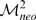 vs 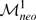), shows a large increase in the number of feedback loops in the former (Table 2). This is intuitively expected from the fact that allosteric regulation allows a local perturbation to spread to remote parts of the network. As a consequence, it has a higher chance of affecting a fast coupling species. This increases the number of non-zero elements in 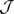, and thus on average the number of feedback loops.

## Discussion

The regulation of gene expression is tightly interwoven with metabolism and signal transduction. A realistic view of gene regulatory networks should therefore not only include direct interactions resulting from transcription regulation, but also indirect regulatory interactions mediated by metabolic effectors, as in the classical example of the *lac* operon [38], [39]. We show here how such a regulatory network can be derived from the network of biochemical reactions in a mathematically rigorous way.

Our approach starts from a model of the biochemical reaction system in the form of Eq. 1. We reformulate this system into coupled fast and slow subsystems, by distinguishing between reactions that are fast and slow in the physiological range of interest, and by redefining fast and slow variables accordingly (Sec. 1 of Text S1). This is rather straightforward to achieve for the types of systems considered here, as enzymatic and complex formation reactions are typically fast on the time-scale of protein synthesis and degradation. Assuming that the fast subsystem is at quasi-steady state, the indirect interactions between genes are now defined by the Jacobian matrix 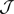 in Eq. 6. In order to derive the indirect interactions between genes by means of this matrix, the rate laws defining the reaction rates do not need to be specified: the dependencies of the reaction rates on metabolite and enzyme concentrations are sufficient. The signs of these partial derivatives are usually unambiguously defined once the metabolic flux directions are fixed. Their substitution into the symbolic expressions of the Jacobian matrix allows the computation of the global effect of a change in gene expression, if such an effect can be unambiguously determined.

The advantage of this approach is that it does not require fully specified kinetic models with numerical values for the parameters, instead of weaker information on the signs of the partial derivatives (see [40] for related ideas in a different context). This information may not be available and the results would be less generic, that is, only hold for these specific kinetic mechanisms and parameter values. Moreover, numerical calculation of 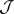 requires the state space of the system to be sampled. For larger models with many variables, this may become very costly. For systems of the size studied in this paper, the derivation of the symbolic expressions does not pose computational problems, although this may change if still larger systems are considered. An interesting topic for further research would be the development of methods that combine symbolic and numerical computations in a clever way.

The derivation of direct and indirect interactions between genes has been addressed before, notably by methods for the inference of networks from transcriptome and other high-throughput data (see [1], [41], [42] for representative examples). Our approach is different from these methods in that it does not infer the interactions from experimental data, but rather starts with available knowledge on the underlying biochemical reaction system. The results are complementary, in the sense that we present a principled way to obtain a core structure of the network that can be completed or refined through data-driven inference procedures. Other related approaches are extensions of flux balance analysis (FBA) that aim at integrating gene regulation with metabolism (*e.g.*, [43]–[45]). Gene regulation is modeled by Boolean rules and, like in our approach, the kinetic rate laws are not specified. The two approaches are quite different though. We do not aim at predicting flux distributions under different environmental conditions, but rather at eliciting indirect interactions between genes mediated by metabolism and to identify modifications of the interaction structure following changes in flux directions. Our approach can thus be seen as a model reduction that uncovers the effective network structure on the time-scale of gene expression. The indirect interactions are expected to have important consequences for the network dynamics, but we leave an analysis of these aspects for further work.

Applied to the carbon assimilation network in *E. coli* our method shows that the resulting gene regulatory network is much more densely connected than the purely transcriptional regulatory network. We notably observe a strong increase of the average connectivity of the network and the number of feedback loops. The indirect interactions revealed by our analysis are operative on the time-scale of gene expression and therefore cannot be ignored. However, some of these may be too weak to be physiologically important, so the actual connectivity may be lower than predicted. In order to decide on the relative strength of the interactions, additional quantitative information is required.

We are not aware of any systematic experimental studies to test the predicted indirect regulatory interactions, with the exception of transcriptome studies using deletion mutants. Notice that these results should be taken with some care for the validation of the derived indirect interactions, as the deletion of a mutant may change the direction of the fluxes and thus the sign of the interactions. In this case, the data agree well with the interaction matrix in Table 1. For instance, our method correctly predicts that a *pykF* deletion leads to increased expression of *fruR* and decreased expression of *cya* during glycolysis [46]. Moreover, in a 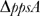 *ppsA* strain the expression of *crp* is lower during gluconeogenesis [47], in agreement with the interaction matrix (Sec. 3 of Text S3).

The most remarkable conclusion of our study of the *E. coli* network is that for given growth conditions, the signs of the indirect interactions are largely independent of the exact form of kinetic rate laws and precise parameter values. The fact that most interactions have an unequivocal sign was not expected on the basis of results obtained with similar approaches for the qualitative analysis of ecological and economic systems [48]–[50]. We have interpreted this surprising finding in terms of sufficient conditions for sign-determinedness. The conditions help us understand what causes most of the interactions in the *E. coli* network to be sign-determined and some of them to be sign-undetermined. The most important of these conditions is the requirement that the concentration control coefficients of the fast coupling species are unambiguously defined. This condition is indeed satisfied by three of the four models studied, but violated by the glycolysis model with allosteric effects, due to the regulation of PykF activity by FBP. The determinate sign of most of the indirect interactions is interesting, because it points at the robustness of the effective structure of this network to changes in the kinetic properties of enzymes and other biochemical reaction species.

Another interesting finding is that radical changes in the environment, *e.g.*, the exhaustion of glucose, may invert the signs of indirect interactions, resulting in a complete rearrangement of the feedback structure of the *E. coli* gene regulatory network. The change in growth conditions affects the direction of the metabolic fluxes, which translates into a switch of the sign of some of the concentration control coefficients. Such an overall modification of the control architecture in response to environmental perturbations may be beneficial to the cell, as it increases its adaptive flexibility. Related to this, radical changes in the genetic background, *e.g.*, the knock-out of a particular gene, may also invert metabolic fluxes and thus change the sign or even the existence of indirect interactions. This may have important consequences for the interpretation of transcriptome data, which often take the form of knock-out datasets [1].

The approach described in this paper provides a sound methodological basis for investigating gene regulatory networks. Its application to *E. coli* carbon assimilation leads to novel insights into the structure of this network. How much of these carry over to other organisms? While the increased connection density and the dependency of the interaction signs on the environmental conditions follow rather straightforwardly from the theory, there is no *a priori* reason why a network should be sign-determined. However, since sign-determinedness confers robustness to the regulatory structure of the system, an important functional requirement [51], it may be more common than expected on purely mathematical grounds.

## Supporting Information

Text S1 Supporting information Text S1.
(0.11 MB PDF)

Text S2 Supporting Information Text S2.
(0.14 MB PDF)

Text S3 Supporting information Text S3.
(0.24 MB PDF)
