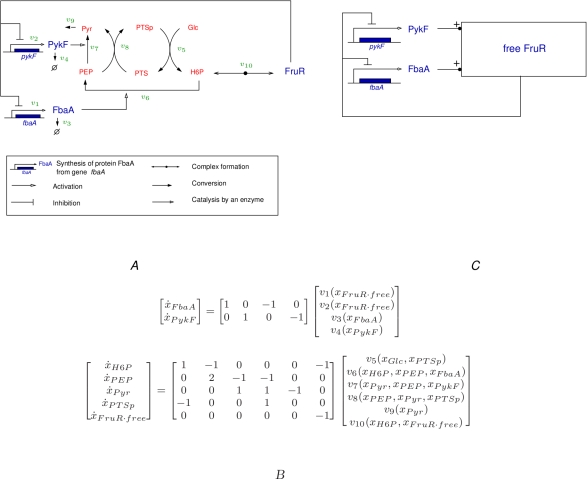# Correction: The Carbon Assimilation Network in *Escherichia coli* Is Densely Connected and Largely Sign-Determined by Directions of Metabolic Fluxes

**DOI:** 10.1371/annotation/ed46bce4-e4e1-4df5-bad3-fac0770efeac

**Published:** 2010-08-03

**Authors:** Valentina Baldazzi, Delphine Ropers, Yves Markowicz, Daniel Kahn, Johannes Geiselmann, Hidde de Jong

Part B of Figure 3 is not in the figure. Please see the corrected Figure 3 here: 

**Figure:**