# Supplementary material for: The Carbon Assimilation Network in Escherichia coli Is Densely Connected and Largely Sign-Determined by Directions of Metabolic Fluxes
Source: PLoS Comput Biol. 2010 Jun 10;6(6):e1000812. doi: 10.1371/journal.pcbi.1000812 (PMC2883603; doi:10.1371/journal.pcbi.1000812)
Supplement: Text S1 — Supporting information Text S1. (0.11 MB PDF) [file pcbi.1000812.s001.pdf]

Supporting Information Text S1 for "The carbon assimilation network in *Escherichia coli* is densely connected and largely sign-determined by directions of metabolic fluxes"

Valentina Baldazzi, Delphine Ropers, Yves Markowicz, Daniel Kahn,
Johannes Geiselmann, Hidde de Jong

Contents

1 Model reduction **1**

2 Sufficient conditions for sign determinedness of network **2**

1 Model reduction

To reduce the model

$$\dot{x} = N\,v(x) \tag{1}$$

we exploit the existence of time-scale hierarchies in the carbon assimilation network [1]. In particular, two different time-scales are distinguished, one corresponding to the slow processes (protein synthesis and degradation) and one to the fast processes (complex formation and enzymatic reactions). In order to better bring out these time-scales in the model, following [1], we divide the rate vector $v \in \mathbb{R}^q$ in a fast and a slow component. More particularly, we define $v^s \in \mathbb{R}^p$ and $v^f \in \mathbb{R}^{q-p}, p < q$, such that $v = [v^s\ v^f]'$, and in the physiological range of interest $v_i^s \ll v_j^f$ for all $1 \leq i \leq p$ and $1 \leq j \leq q-p$. Protein synthesis and degradation are typical slow reactions, while enzymatic and complex formation reactions are typically fast.

The separation of fast and slow reactions motivates a linear transformation $T \in \mathbb{Z}^n \times \mathbb{Z}^n$ of the variables:

$$\begin{bmatrix} x^s \\ x^f \end{bmatrix} = T\,x, \tag{2}$$

such that

$$\begin{bmatrix} N^s & 0 \\ N^{s'} & N^f \end{bmatrix} = T\,N. \tag{3}$$

We call $x^s \in \mathbb{R}_+^m$ and $x^f \in \mathbb{R}_+^{n-m}$ slow and fast variables of the system, respectively ($m < n$). $N^s \in \mathbb{Z}^m \times \mathbb{Z}^p$ and $N^{s'} \in \mathbb{Z}^{n-m} \times \mathbb{Z}^p$ are stoichiometry matrices for the slow reactions, and $N^f \in \mathbb{Z}^{n-m} \times \mathbb{Z}^{q-p}$ is a stoichiometry matrix for the fast reactions. The slow variables typically correspond to total protein concentrations, whereas the fast variables include concentrations

of metabolites and biochemical complexes. The variable transformation allows the original system Eq. 1 to be rewritten as:

$$\dot{x}^s = N^s\, v^s(x^s, x^f), \qquad x^s(0) = x^s_0, \tag{4}$$

$$\dot{x}^f = N^{s'}\, v^s(x^s, x^f) + N^f\, v^f(x^s, x^f) \approx N^f\, v^f(x^s, x^f), \quad x^f(0) = x^f_0. \tag{5}$$

Notice that the slow variables are only influenced by slow reactions, while the fast reactions dominate the change of the fast variables. The effect of slow rates is neglected in the equations for fast variables, as $v^s(x^s, x^f) \ll v^f(x^s, x^f)$ in the physiological range of interest.

In the main text we have assumed that N has been reduced for conserved quantities, following the approach in [2]. Notice that, under this assumption, the stoichiometry matrices in Eqs. 4 and 5 are full rank and, therefore, the Jacobian matrix of the fast system, called M in the main text, is non-singular.

As summarized in the main text, and explained in detail in [1], the separation of the system into a slow and a fast part allows the application of the quasi-steady-state approximation $\dot{x}^f = 0$, implying

$$N^f\, v^f(x^s, x^f) = 0. \tag{6}$$

It is sometimes possible to decompose the fast system into independent subsystems, each corresponding to a block in the stoichiometry matrix:

$$N^f\, v^f(x^f, x^s) = \begin{bmatrix} N^f_1 & \cdots & 0 \\ \vdots & \ddots & \vdots \\ 0 & \cdots & N^f_r \end{bmatrix} \begin{bmatrix} v^f_1(x^f_1, x^s) \\ \vdots \\ v^f_r(x^f_r, x^s) \end{bmatrix}. \tag{7}$$

This means that the system of algebraic equations 6 resulting from the quasi-steady-state approximation decomposes into r independent subsystems. This greatly simplifies the application of the method presented in the main text.

2 Sufficient conditions for sign determinedness of network

We define sign-determinedness of a gene regulatory network as the uniqueness of the signs of the elements of the Jacobian matrix $\mathcal{J}$ of the slow system, given by Eq. 6 of the main text, independently of the exact form of kinetic rate laws and precise parameter values. In the Methods section we enumerate four sufficient conditions for the sign-determinedness of the reconstructed network at the slow time-scale, which we briefly repeat here.

C1 A slow variable acts directly either on the slow system or on the fast system, but not on both simultaneously.

C2 No variable has direct antagonistic (*i.e.*, both activating and inhibiting) effects on a slow variable.

C3 The concentration control coefficients of the fast coupling species with respect to the slow variables have a determinate sign.

C4 If a slow variable contributes to the concentration control of several fast coupling species, the latter do not simultaneously regulate any of the slow variables (no concerted regulation).

We demonstrate the following proposition:

Prop. 1 Under conditions **C1**-**C4**, the network structure defined by the Jacobian matrix $\mathcal{J}$ is sign-determined.

Proof. The element of Eq. 6 of the main text describing the effect of x_j^s on x_i^s reads

$$\frac{\partial \dot{x}_i^s}{\partial x_j^s} = \sum_k n_{ik}^s \frac{\partial v_k^s(x^s, x^f)}{\partial x_j^s} + \sum_m \Big[\sum_k n_{ik}^s \frac{\partial v_k^s(x^s, x^f)}{\partial x_m^f}\Big] \frac{\partial g_m(x^s)}{\partial x_j^s} \tag{8}$$

where $N^s = \{n_{ik}^s\}$ is the stoichiometry matrix for the slow subsystem (see Sec. 1). Remember that the sign of the partial derivatives $\partial v^s(x^s, x^f)/\partial x^s$ and $\partial v^s(x^s, x^f)/\partial x^f$ is generally known from the literature, as discussed in the Methods section. In this context, conditions **C1**-**C4** provide additional constraints on the sign of the terms in Eq. 8, such that the resulting Jacobian is sign-determined.

Condition **C1** imposes that for all j, either $\partial v_k^s(x^s, x^f)/\partial x_j^s = 0$, for all k, or $\partial g_m(x^s)/\partial x_j^s = 0$, for all m. This means that only one of the summation terms in Eq. 8 is non-zero, thus excluding an antagonism between the direct and the indirect regulations exerted by a slow variable. The remaining conditions then assure that this non-zero term has a determinate sign, as shown below.

Under condition **C2**, all terms in the sum $\sum_k n_{ik}^s \, \partial v_k^s(x^s, x^f)/\partial x_j^s$ in Eq. 8 have the same sign (positive, negative, zero). Idem for the sum $\sum_k n_{ik}^s \, \partial v_k^s(x^s, x^f)/\partial x_m^f$, for all m. This condition is sufficient to fix the sign of the first summation term in Eq. 8, and thus of the direct interaction between x_j^s and x_i^s.

Conditions **C3** and **C4** deal with the second summation term of Eq. 8, and thus with the sign of the indirect interaction. We start with condition **C4**: it states that the second summation term of Eq. 8 reduces to

$$\Big[\sum_k n_{ik}^s \frac{\partial v_k^s(x^s, x^f)}{\partial x_{\bar{m}}^f}\Big] \frac{\partial g_{\bar{m}}(x^s)}{\partial x_j^s} \tag{9}$$

for some $\bar{m}$. Moreover, condition **C3** guarantees that the control coefficient $\partial g_{\bar{m}}(x^s)/\partial x_j^s$ has a determinate sign. As a consequence, in conjunction with **C2**, the sign of Eq. 9, and thus of the second summation term in Eq. 8, is unambiguous. This fixes the sign of the indirect interaction between x_j^s and x_i^s. In conclusion, the four conditions are sufficient to guarantee the sign-determinedness of the the Jacobian matrix $\mathcal{J}$, and thus of the reconstructed network. □

Remember that **C1**-**C4** are only sufficient conditions and that sign-determinedness can be obtained even when some of the conditions are violated.

References

1. Heinrich R, Schuster S (1996) The Regulation of Cellular Systems. New York: Chapman & Hall.

2. Reder C (1988) Metabolic control theory: a structural approach. J Theor Biol 135: 175–201.
