# Supplementary material for: The Carbon Assimilation Network in Escherichia coli Is Densely Connected and Largely Sign-Determined by Directions of Metabolic Fluxes
Source: PLoS Comput Biol. 2010 Jun 10;6(6):e1000812. doi: 10.1371/journal.pcbi.1000812 (PMC2883603; doi:10.1371/journal.pcbi.1000812)
Supplement: Text S2 — Supporting Information Text S2. (0.14 MB PDF) [file pcbi.1000812.s002.pdf]

Supporting Information Text S2 for "The carbon assimilation network in *Escherichia coli* is densely connected and largely sign-determined by directions of metabolic fluxes"

Valentina Baldazzi, Delphine Ropers, Yves Markowicz, Daniel Kahn, Johannes Geiselmann, Hidde de Jong

1 Analysis of the simplified glycolysis model

We present the complete analysis of the example in Fig. 3 of the main text, following the method introduced previously. The model is a simplified representation of the glycolysis pathway, during growth on glucose, including regulation on both the metabolic and genetic levels. It is defined by the following system of ODEs, written in the form of Eqs. 3 and 4 of the main text, that is, with the separation of fast and slow variables:

$$\begin{bmatrix}\dot{x}_{FbaA}\\ \dot{x}_{PykF}\end{bmatrix}=\begin{bmatrix}1 & 0 & -1 & 0\\ 0 & 1 & 0 & -1\end{bmatrix}\begin{bmatrix}v_1(x_{FruR\cdot free})\\ v_2(x_{FruR\cdot free})\\ v_3(x_{FbaA})\\ v_4(x_{PykF})\end{bmatrix} \qquad (1)$$

$$\begin{bmatrix}\dot{x}_{H6P}\\ \dot{x}_{PEP}\\ \dot{x}_{Pyr}\\ \dot{x}_{PTSp}\\ \dot{x}_{FruR\cdot free}\end{bmatrix}=\begin{bmatrix}1 & -1 & 0 & 0 & 0 & -1\\ 0 & 2 & -1 & -1 & 0 & 0\\ 0 & 0 & 1 & 1 & -1 & 0\\ -1 & 0 & 0 & 1 & 0 & 0\\ 0 & 0 & 0 & 0 & 0 & -1\end{bmatrix}\begin{bmatrix}v_5(x_{Glc},x_{PTSp})\\ v_6(x_{H6P},x_{PEP},x_{FbaA})\\ v_7(x_{Pyr},x_{PEP},x_{PykF})\\ v_8(x_{PEP},x_{Pyr},x_{PTSp})\\ v_9(x_{Pyr})\\ v_{10}(x_{H6P},x_{FruR\cdot free})\end{bmatrix} \qquad (2)$$

where $x^s=[x_{FbaA},x_{PykF}]'$ and $x^f=[x_{H6P},x_{PEP},x_{Pyr},x_{PTSp},x_{FruR\cdot free}]'$.

The objective is to estimate the signs of the element of the Jacobian matrix of the slow system by means of Eqs. 6 and 8 of the main text. Fig. 1 summarizes the approach in a schematic way, while Fig. 2 illustrates the computation on the example network.

1. **Compute the Jacobian matrix M for the fast system and its inverse M^{-1}.** For the model in Fig. 3 of the main text, we compute

$$M=\begin{bmatrix}-\frac{\partial v_6}{\partial x_{H6P}}-\frac{\partial v_{10}}{\partial x_{H6P}} & -\frac{\partial v_6}{\partial x_{PEP}} & 0 & \frac{\partial v_5}{\partial x_{PTSp}} & -\frac{\partial v_{10}}{\partial x_{FruR\cdot free}}\\ 2\frac{\partial v_6}{\partial x_{H6P}} & 2\frac{\partial v_6}{\partial x_{PEP}}-\frac{\partial v_7}{\partial x_{PEP}}-\frac{\partial v_8}{\partial x_{PEP}} & -\frac{\partial v_8}{\partial x_{Pyr}} & -\frac{\partial v_8}{\partial x_{PTSp}} & 0\\ 0 & \frac{\partial v_7}{\partial x_{PEP}}+\frac{\partial v_8}{\partial x_{PEP}} & \frac{\partial v_8}{\partial x_{Pyr}}-\frac{\partial v_9}{\partial x_{Pyr}} & \frac{\partial v_8}{\partial x_{PTSp}} & 0\\ 0 & \frac{\partial v_8}{\partial x_{PEP}} & \frac{\partial v_8}{\partial x_{Pyr}} & \frac{\partial v_8}{\partial x_{PTSp}}-\frac{\partial v_5}{\partial x_{PTSp}} & 0\\ -\frac{\partial v_{10}}{\partial x_{H6P}} & 0 & 0 & 0 & -\frac{\partial v_{10}}{\partial x_{FruR\cdot free}}\end{bmatrix} \qquad (3)$$

where the partial derivatives have the sign indicated in Table 1, according to the convention of positive fluxes in the glycolytic direction. Using the Symbolic Math Toolbox of MATLAB (MathWorks), we compute

$$M^{-1} = \frac{1}{(2\sigma_4 + \sigma_3 + \sigma_2 - \sigma_1)} \cdot \begin{bmatrix} m_{11}^{-1} & m_{12}^{-1} & m_{13}^{-1} & m_{14}^{-1} & 0 \\ m_{21}^{-1} & m_{22}^{-1} & m_{23}^{-1} & m_{24}^{-1} & 0 \\ m_{31}^{-1} & m_{32}^{-1} & m_{33}^{-1} & m_{34}^{-1} & 0 \\ m_{41}^{-1} & m_{42}^{-1} & m_{43}^{-1} & m_{44}^{-1} & 0 \\ m_{51}^{-1} & m_{52}^{-1} & m_{53}^{-1} & m_{54}^{-1} & m_{55}^{-1} \end{bmatrix} \tag{4}$$

where

$$m_{11}^{-1} = (2\sigma_7 - 2\sigma_6 + \sigma_1 + \sigma_3 + 2\sigma_5 - \sigma_2)/\frac{\partial v_6}{\partial x_{H6P}} \tag{5}$$

$$m_{12}^{-1} = (\sigma_3 + \sigma_7 + \sigma_5 - \sigma_6 + \sigma_4)/\frac{\partial v_6}{\partial x_{H6P}} \tag{6}$$

$$m_{13}^{-1} = -\frac{\partial v_5}{\partial x_{PTSp}}\frac{\partial v_8}{\partial x_{Pyr}}(\frac{\partial v_6}{\partial x_{PEP}} - \frac{\partial v_7}{\partial x_{PEP}})/\frac{\partial v_6}{\partial x_{H6P}} \tag{7}$$

$$m_{14}^{-1} = (2\sigma_7 - 2\sigma_6 + \sigma_1 + \sigma_3 + \sigma_5)/\frac{\partial v_6}{\partial x_{H6P}} \tag{8}$$

$$m_{21}^{-1} = -2(\frac{\partial v_8}{\partial x_{Pyr}}\frac{\partial v_5}{\partial x_{PTSp}} + \frac{\partial v_9}{\partial x_{Pyr}}\frac{\partial v_8}{\partial x_{PTSp}} - \frac{\partial v_9}{\partial x_{Pyr}}\frac{\partial v_5}{\partial x_{PTSp}}) \tag{9}$$

$$m_{22}^{-1} = -(\frac{\partial v_8}{\partial x_{Pyr}}\frac{\partial v_5}{\partial x_{PTSp}} + \frac{\partial v_9}{\partial x_{Pyr}}\frac{\partial v_8}{\partial x_{PTSp}} - \frac{\partial v_9}{\partial x_{Pyr}}\frac{\partial v_5}{\partial x_{PTSp}}) \tag{10}$$

$$m_{23}^{-1} = \frac{\partial v_5}{\partial x_{PTSp}}\frac{\partial v_8}{\partial x_{Pyr}} \tag{11}$$

$$m_{24}^{-1} = -(2\frac{\partial v_8}{\partial x_{Pyr}}\frac{\partial v_5}{\partial x_{PTSp}} - 2\frac{\partial v_9}{\partial x_{Pyr}}\frac{\partial v_5}{\partial x_{PTSp}} + \frac{\partial v_9}{\partial x_{Pyr}}\frac{\partial v_8}{\partial x_{PTSp}}) \tag{12}$$

$$m_{31}^{-1} = -2(-\frac{\partial v_8}{\partial x_{PEP}}\frac{\partial v_5}{\partial x_{PTSp}} + \frac{\partial v_7}{\partial x_{PEP}}\frac{\partial v_8}{\partial x_{PTSp}} - \frac{\partial v_7}{\partial x_{PEP}}\frac{\partial v_5}{\partial x_{PTSp}}) \tag{13}$$

$$m_{32}^{-1} = -(-\frac{\partial v_8}{\partial x_{PEP}}\frac{\partial v_5}{\partial x_{PTSp}} + \frac{\partial v_7}{\partial x_{PEP}}\frac{\partial v_8}{\partial x_{PTSp}} - \frac{\partial v_7}{\partial x_{PEP}}\frac{\partial v_5}{\partial x_{PTSp}}) \tag{14}$$

$$m_{33}^{-1} = -(\frac{\partial v_8}{\partial x_{PEP}}\frac{\partial v_5}{\partial x_{PTSp}} - \frac{\partial v_7}{\partial x_{PEP}}\frac{\partial v_5}{\partial x_{PTSp}} + \frac{\partial v_7}{\partial x_{PEP}}\frac{\partial v_8}{\partial x_{PTSp}}) \tag{15}$$

$$m_{34}^{-1} = 2\frac{\partial v_5}{\partial x_{PTSp}}(\frac{\partial v_7}{\partial x_{PEP}} + \frac{\partial v_8}{\partial x_{PEP}}) \tag{16}$$

$$m_{41}^{-1} = 2(\frac{\partial v_8}{\partial x_{PEP}}\frac{\partial v_9}{\partial x_{Pyr}} + \frac{\partial v_8}{\partial x_{Pyr}}\frac{\partial v_7}{\partial x_{PEP}}) \tag{17}$$

$$m_{42}^{-1} = \frac{\partial v_8}{\partial x_{PEP}}\frac{\partial v_9}{\partial x_{Pyr}} + \frac{\partial v_8}{\partial x_{Pyr}}\frac{\partial v_7}{\partial x_{PEP}} \tag{18}$$

$$m_{43}^{-1} = \frac{\partial v_8}{\partial x_{Pyr}}\frac{\partial v_7}{\partial x_{PEP}} \tag{19}$$

$$m_{44}^{-1} = \frac{\partial v_9}{\partial x_{Pyr}}(\frac{\partial v_7}{\partial x_{PEP}} + \frac{\partial v_8}{\partial x_{PEP}}) \tag{20}$$

$$m_{51}^{-1} = -(2\sigma_7 - 2\sigma_6 + \sigma_1 + \sigma_3 + 2\sigma_5 - \sigma_2)\frac{\partial v_{10}}{\partial x_{H6P}}/\frac{\partial v_6}{\partial x_{H6P}}/\frac{\partial v_{10}}{\partial x_{FruR\cdot free}} \tag{21}$$

$$m_{52}^{-1} = -(\sigma_3 + \sigma_7 + \sigma_5 - \sigma_6 + \sigma_4)\frac{\partial v_{10}}{\partial x_{H6P}}/\frac{\partial v_6}{\partial x_{H6P}}/\frac{\partial v_{10}}{\partial x_{FruR\cdot free}} \tag{22}$$

$$m_{53}^{-1} = \frac{\partial v_8}{\partial x_{Pyr}}\frac{\partial v_5}{\partial x_{PTSp}}\frac{\partial v_{10}}{\partial x_{H6P}}(\frac{\partial v_6}{\partial x_{PEP}} - \frac{\partial v_7}{\partial x_{PEP}})/\frac{\partial v_6}{\partial x_{H6P}}/\frac{\partial v_{10}}{\partial x_{FruR\cdot free}} \tag{23}$$

$$m_{54}^{-1} = -(2\sigma_7 - 2\sigma_6 + \sigma_1 + \sigma_3 + \sigma_5)\frac{\partial v_{10}}{\partial x_{H6P}}/\frac{\partial v_6}{\partial x_{H6P}}/\frac{\partial v_{10}}{\partial x_{FruR\cdot free}} \tag{24}$$

$$m_{55}^{-1} = -(2\sigma_4 + \sigma_3 + \sigma_2 - \sigma_1)/\frac{\partial v_{10}}{\partial x_{FruR\cdot free}} \tag{25}$$

and

$$
\begin{aligned}
\sigma_1 &= \frac{\partial v_9}{\partial x_{Pyr}} \frac{\partial v_5}{\partial x_{PTSp}} \frac{\partial v_7}{\partial x_{PEP}} && (26)\\
\sigma_2 &= \frac{\partial v_9}{\partial x_{Pyr}} \frac{\partial v_8}{\partial x_{PTSp}} \frac{\partial v_7}{\partial x_{PEP}} && (27)\\
\sigma_3 &= \frac{\partial v_9}{\partial x_{Pyr}} \frac{\partial v_5}{\partial x_{PTSp}} \frac{\partial v_8}{\partial x_{PEP}} && (28)\\
\sigma_4 &= \frac{\partial v_8}{\partial x_{Pyr}} \frac{\partial v_7}{\partial x_{PEP}} \frac{\partial v_5}{\partial x_{PTSp}} && (29)\\
\sigma_5 &= \frac{\partial v_9}{\partial x_{Pyr}} \frac{\partial v_6}{\partial x_{PEP}} \frac{\partial v_8}{\partial x_{PTSp}} && (30)\\
\sigma_6 &= \frac{\partial v_9}{\partial x_{Pyr}} \frac{\partial v_6}{\partial x_{PEP}} \frac{\partial v_5}{\partial x_{PTSp}} && (31)\\
\sigma_7 &= \frac{\partial v_8}{\partial x_{Pyr}} \frac{\partial v_6}{\partial x_{PEP}} \frac{\partial v_5}{\partial x_{PTSp}} && (32)
\end{aligned}
$$

2. **Determine $N^f \frac{\partial v^f}{\partial x^s}$ for each slow variable.** As an example, we show the case of x_{PykF}:

$$
N^f \frac{\partial v^f}{\partial x_{PykF}} = \begin{bmatrix} 0 \\ -\frac{\partial v_7}{\partial x_{PykF}} \\ \frac{\partial v_7}{\partial x_{PykF}} \\ 0 \\ 0 \end{bmatrix} \tag{33}
$$

3. **Compute $\frac{\partial g(x^s)}{\partial x^s}$ using Eq. 8 in the main text.** As above, we illustrate this by means of x_{PykF}.

$$
\frac{\partial g(x^s)}{\partial x_{PykF}} = \frac{1}{(2\sigma_4 + \sigma_3 + \sigma_2 - \sigma_1)} \cdot \tag{34}
$$

$$
\begin{bmatrix}
\frac{\partial v_7}{\partial x_{PykF}} (\sigma_3 + 2\,\sigma_7 + \sigma_5 - \sigma_6) / \frac{\partial v_6}{\partial x_{H6P}} \\
-\frac{\partial v_7}{\partial x_{PykF}} (2 \frac{\partial v_8}{\partial x_{Pyr}} \frac{\partial v_5}{\partial x_{PTSp}} + \frac{\partial v_9}{\partial x_{Pyr}} \frac{\partial v_8}{\partial x_{PTSp}} - \frac{\partial v_9}{\partial x_{Pyr}} \frac{\partial v_5}{\partial x_{PTSp}}) \\
2 \frac{\partial v_7}{\partial x_{PykF}} \frac{\partial v_8}{\partial x_{PEP}} \frac{\partial v_5}{\partial x_{PTSp}} \\
\frac{\partial v_7}{\partial x_{PykF}} \frac{\partial v_8}{\partial x_{PEP}} \frac{\partial v_9}{\partial x_{Pyr}} \\
-\frac{\partial v_{10}}{\partial x_{H6P}} \frac{\partial v_7}{\partial x_{PykF}} (\sigma_3 + 2\sigma_7 + \sigma_5 - \sigma_6) / \frac{\partial v_6}{\partial x_{H6P}} / \frac{\partial v_{10}}{\partial x_{FruR \cdot free}}
\end{bmatrix}
$$

The sign of the expression in Eq. 34 depends on the sign of $(2\sigma_4 + \sigma_3 + \sigma_2 - \sigma_1)$, which can be expressed in terms of the coefficients of the characteristic polynomial of M. Let $x^5 + a_1 x^4 + a_2\, x^3 + a_3\, x^2 + a_4\, x + a_5 = 0$ be the characteristic polynomial, where a_5 is the determinant of M. Then, $(2\sigma_4 + \sigma_3 + \sigma_2 - \sigma_1) = -a_5 / (\frac{\partial v_{10}}{\partial x_{FruR \cdot free}} \frac{\partial v_6}{\partial x_{H6P}})$. Given the signs of $\partial v_{10}/\partial x_{FruR \cdot free}$ and $\partial v_6/\partial x_{H6P}$ in Table 1 and the stability condition for the fast system, which imposes $a_5 > 0$, we find $(2\sigma_4 + \sigma_3 + \sigma_2 - \sigma_1) < 0$. We thus infer that $\partial x_{FruR \cdot free}/\partial x_{PykF}$, the last element of the computed vector of symbolic expressions, has a fixed positive sign, regardless of the parameters values and the mathematical form of rate laws (Fig. 2).

4. **Compute $N^s \frac{\partial v^s}{\partial x^s}$ and $N^s \frac{\partial v^s}{\partial x^f}$.**

In the model example, there are no direct transcriptional regulations between the two genes *fbaA* and *pykF*, so that for all x^s

$$N^s \frac{\partial v^s}{\partial x^s} = 0. \tag{35}$$

Slow fluxes depend instead on the fast variable $x_{FruR \cdot free}$, which couples metabolism to gene regulation:

$$N^s \frac{\partial v^s}{\partial x^f} = \begin{bmatrix} 0 & 0 & 0 & 0 & \frac{\partial v_1^s}{\partial x_{FruR \cdot free}} \\ 0 & 0 & 0 & 0 & \frac{\partial v_2^s}{\partial x_{FruR \cdot free}} \end{bmatrix}. \tag{36}$$

5. **Reconstruct the signs of the Jacobian matrix $\mathcal{J}$ for the slow system.** Using Eq. 6 in the main text and the results obtained in steps 3 and 4, we obtain

$$\mathcal{J} = \begin{bmatrix} \frac{\partial \dot{x}_{FbaA}}{\partial x_{FbaA}} & \frac{\partial \dot{x}_{FbaA}}{\partial x_{PykF}} \\ \frac{\partial \dot{x}_{PykF}}{\partial x_{FbaA}} & \frac{\partial \dot{x}_{PykF}}{\partial x_{PykF}} \end{bmatrix} \text{ and } \mathrm{sign}(\mathcal{J}) = \begin{bmatrix} - & - \\ - & - \end{bmatrix}. \tag{37}$$

That is, the sign of $\partial \dot{x}_{FbaA} / \partial x_{PykF}$ is negative (Fig. 2). The complete network is shown in Fig. 3*C* of the main text.

**Protein synthesis and degradation**			
$v_1(x_{FruR\cdot free})$	$\frac{\partial v_1}{\partial x_{FruR\cdot free}} < 0$		
$v_2(x_{FruR\cdot free})$	$\frac{\partial v_2}{\partial x_{FruR\cdot free}} < 0$		
$v_3(x_{FbaA})$	$\frac{\partial v_3}{\partial x_{FbaA}} > 0$		
$v_4(x_{PykF})$	$\frac{\partial v_4}{\partial x_{PykF}} > 0$		
**Enzymatic reactions and complex formation**			
$v_5(x_{PTSp})$	$\frac{\partial v_5}{\partial x_{PTSp}} > 0$		
$v_6(x_{FbaA}, x_{H6P}, x_{PEP})$	$\frac{\partial v_6}{\partial x_{FbaA}} > 0$	$\frac{\partial v_6}{\partial x_{H6P}} > 0$	$\frac{\partial v_6}{\partial x_{PEP}} < 0$
$v_7(x_{PykF}, x_{PEP})$	$\frac{\partial v_7}{\partial x_{PykF}} > 0$	$\frac{\partial v_7}{\partial x_{PEP}} > 0$	
$v_8(x_{PEP}, x_{Pyr}, x_{PTSp})$	$\frac{\partial v_8}{\partial x_{Pyr}} < 0$	$\frac{\partial v_8}{\partial x_{PEP}} > 0$	$\frac{\partial v_8}{\partial x_{PTSp}} < 0$
$v_9(x_{Pyr})$	$\frac{\partial v_9}{\partial x_{Pyr}} > 0$		
$v_{10}(x_{H6P}, x_{FruR\cdot free})$	$\frac{\partial v_{10}}{\partial x_{H6P}} > 0$	$\frac{\partial v_{10}}{\partial x_{FruR\cdot free}} > 0$	

Table 1: **Fluxes and derivative signs for the simplified glycolysis example.**

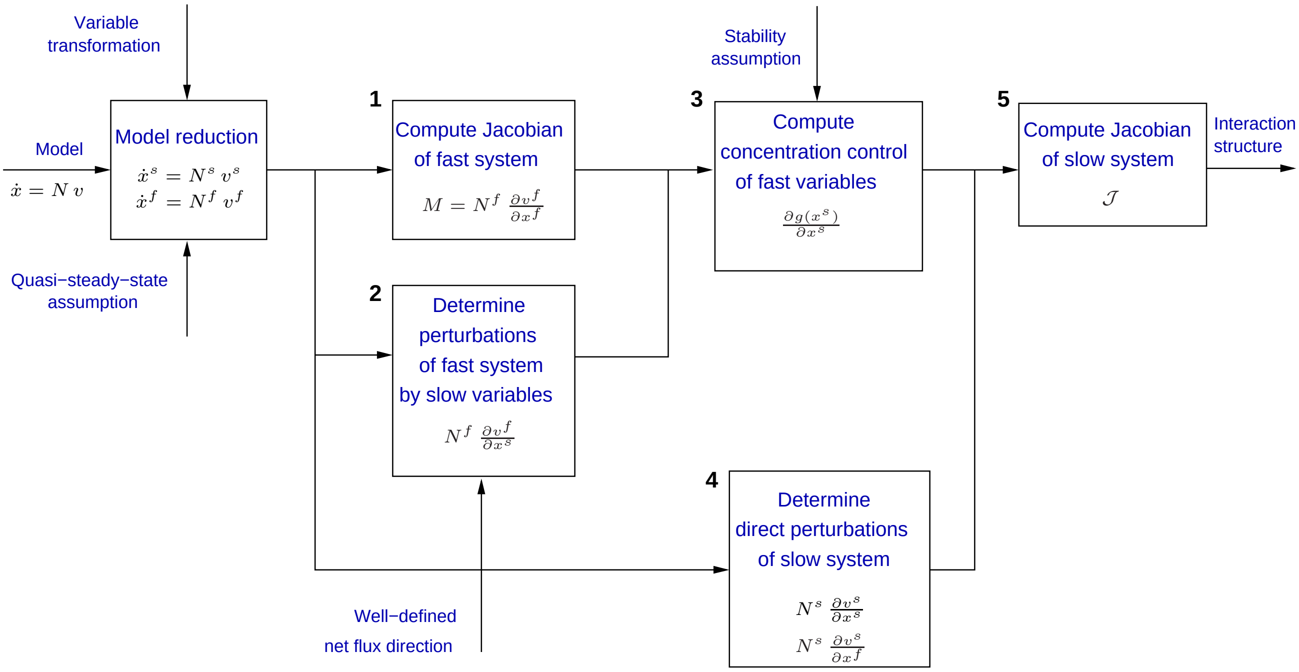

Figure 1: **Flowchart of the derivation of direct and indirect interactions**, from the kinetic model in Eq. 1 to the Jacobian matrix in Eq. 6 of the main text. The numbers next to the boxes correspond to the steps in Text S2. Notice that the example system in Fig. 3*B* is already written in the reduced form, with separated fast and slow variables.

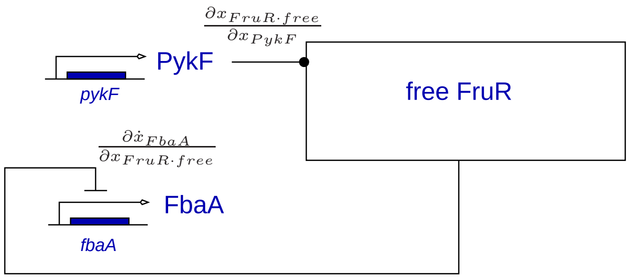

Figure 2: **Schematic representation of the computation of $\partial \dot{x}_{FbaA}/\partial x_{PykF}$, that is, the influence of PykF on the expression of the gene *fbaA*.** The partial derivative is decomposed as the product of $\partial \dot{x}_{FbaA}/\partial x_{FruR\cdot free}$ and $\partial x_{FruR\cdot free}/\partial x_{PykF}$, accounting for the regulation of *fbaA* by free FruR and the influence of the glycolytic enzyme PykF on the free FruR level, respectively. The box labeled 'free FruR' represents the metabolic network mediating the latter influence.
