# Supplementary material for: The Carbon Assimilation Network in Escherichia coli Is Densely Connected and Largely Sign-Determined by Directions of Metabolic Fluxes
Source: PLoS Comput Biol. 2010 Jun 10;6(6):e1000812. doi: 10.1371/journal.pcbi.1000812 (PMC2883603; doi:10.1371/journal.pcbi.1000812)
Supplement: Text S3 — Supporting information Text S3. (0.24 MB PDF) [file pcbi.1000812.s003.pdf]

Supporting Information Text S3 for "The carbon assimilation network in *Escherichia coli* is densely connected and largely sign-determined by directions of metabolic fluxes"

Valentina Baldazzi, Delphine Ropers, Yves Markowicz, Daniel Kahn, Johannes Geiselmann, Hidde de Jong

Contents

1 Complete carbon assimilation model **1**

2 Reduced carbon assimilation model **2**

2.1 Glycolysis model 2

2.2 Gluconeogenesis model 3

3 Derivation of interaction matrix for the reduced carbon assimilation model **3**

3.1 Results for the gluconeogenesis model 4

3.2 Results for the glycolysis model 4

4 Feedback loops in carbon assimilation network **6**

1 Complete carbon assimilation model

We specify here the complete integrated model introduced in the Results section of the main text, describing the regulatory control of glycolysis/gluconeogenesis in *E. coli.* The metabolic part is essentially based on previous models by Bettenbrock *et al.* [3], Kremling *et al* [4] and Chassagnole *et al.* [5] which we complete with few additional biochemical reactions, such as the one for pyruvate uptake. The pentose-phosphate pathway (PPP) is not explicitly described in the model but we take into account that a small pool of G6P escapes the upper part of glycolysis. The part of the G6P flux that enter PPP amounts to about a fifth of the G6P flux, when *E. coli* cells are grown on minimal medium with glucose [8], and four fifths of the G6P flux when cells are grown on pyruvate. The concentrations of the cofactors (ATP, NADP, etc.) are assumed to be constant and considered as parameters in the present model.

The network controlling the expression of the global regulators is based on Ropers *et al.* [6, 7]. It includes the control of the DNA supercoiling level, the accumulation of the sigma factor RpoS and the Crp·cAMP complex, and it is extended with the addition of the fructose repressor FruR. The genetic regulation of glycolytic and gluconeogenic enzymes by the Crp·cAMP complex and FruR establishes a connection between the genetic and the metabolic parts of the system. All information on the transcriptional regulation of the considered genes is amply documented in curated databases [9] and in related literature (see Table 2). Unless

there is evidence to the contrary, as for instance in the case of RpoS, protein degradation rates are assumed not to be specifically regulated.

Changes in the external environment (*e.g.*, in glucose concentration) are sensed via the PEP:phosphotransferase system (PTS). The PTS system is described in a simplified way, following [4]: the four PTS proteins are represented as a single component PTS, whose phosphorylation state is considered as representative for the phosphorylation state of the protein EIIA^{glc}. In our model, the phosphorylated PTS is considered as an activator of Cya, the enzyme catalyzing the synthesis of the regulatory effector cAMP.

The resulting model has the general form of Eq. 1 of the main text and includes several variables of different nature, *i.e.* proteins, metabolites and complexes, which are listed in Table 1. The functions $v(x)$ describing the reaction rates are not explicitly defined, but the dependencies of the rate laws on specific variables are provided. For example, the reaction catalyzed by the pyruvate kinase (PykF), converting PEP into pyruvate, is indicated as the reaction rate $v_{53}(x_{10}, x_{30}, x_{24})$. It depends on the concentration of PykF (x_{10}), of its substrate PEP (x_{30}) and on the concentration of its allosteric effector FBP (x_{24}). The signs of partial derivatives $\partial v(x)/\partial x$ are also given, according to the convention of positive fluxes in the glycolytic direction (Table 2). Therefore, $\partial v_{53}/\partial x_{10}$ is positive, as an increase in the enzyme concentration leads to an increase of the reaction rate in the glycolytic direction. The same is true for $\partial v_{53}/\partial x_{30} > 0$. The effect of the allosteric regulation is also specified by the sign of the corresponding partial derivative. In particular, FBP is an allosteric activator of PykF activity and thus $\partial v_{53}/\partial x_{24}$ is positive.

2 Reduced carbon assimilation model

The reduction of the carbon assimilation model requires a preliminary definition of fast and slow variables, based on the identification of fast and slow reactions (see Sec. 1 of Text S1). The transformation matrix T is too big to be shown here, so we report the fast and slow variables in tabular form (Table 4). With the above definition of the variables, our system of equations can be rewritten as two subsystems, a slow and a fast one, summarized in Table 5. In doing this we made the following assumptions. The concentrations of DHAP and G3P are lumped together in a single variable, the total amount of PTS is considered constant and by convention we take its phosphorylated form as an independent variable. We thus obtain an ODE system consisting of 21 slow variables and 17 fast variables. Four of the slow variables can be eliminated, by assuming that the concentrations of constitutively expressed enzymes (Pgi, Fbp, TpiA, and GpmI) are constant and fixed at the steady-state value.

We apply the QSS approximation to the reformulated system, by putting the time derivative of fast variables equal to 0. This results in a system of algebraic equations that we further simplify by distinguishing between two possible growth conditions for the bacteria, either growth on glucose or on pyruvate. In each growth condition, we fix a net direction for the metabolic fluxes (see Methods section in the main text), and we thus obtain two distinct models for glycolysis and gluconeogenesis.

2.1 Glycolysis model

During growth on glucose, net fluxes flow from G6P to pyruvate [10], and to the TCA cycle. Glucose uptake is performed via the PTS system and is described by a rate (v_{65}) that depends on the PTSp concentration [10]. Some of the fluxes, specific for growth on pyruvate, such as

those arising from the reactions catalyzed by phosphoenolpyruvate synthase (PpsA), and by fructose-biphosphatase (Fbp), are negligible during glycolysis. The corresponding fluxes (v_{54} and v_{46}, respectively) are put to 0. Moreover, the variables corresponding to the enzymes (x^s_{11} for PpsA) and (x^s_3 for Fbp) are omitted from the model. This yields the reduced glycolysis model $\mathcal{M}^2_{glyco}$.

Equations for model $\mathcal{M}^1_{glyco}$, excluding allosteric effects, are the same as those for $\mathcal{M}^2_{glyco}$, but the fluxes $v_{53}(x^s_{10}, x^f_8, x^f_3)$ and $v_{45}(x^s_2, x^f_2, x^f_8)$ are now considered to be independent of the concentrations of FBP (x^f_3) and PEP (x^f_8), respectively. That is, $\partial v_{53}/\partial x^f_3$ and $\partial v_{45}/\partial x^f_8$ are set to 0.

2.2 Gluconeogenesis model

When glucose is depleted, *E. coli* starts to take up alternative carbon sources such as organic acids [10]. Model $\mathcal{M}_{neo}$ considers the case in which pyruvate is employed as the main carbon source and converted to G6P via the gluconeogenic pathway. Similarly to the glycolytic model, we do not consider fluxes that are negligible during growth on pyruvate. As a result, the fluxes of reactions catalyzed by phosphofructokinase (PfkA) and pyruvate kinase (PykF) (v_{45} and v_{53}, respectively) are set to 0. Moreover, the variables corresponding to the enzymes (x^s_2 for PfkA) and (x^s_{10} for PykF) are omitted from the model. The glucose uptake flux v_{65} and the pyruvate export flux v_{63} are also set to 0. This yields the reduced glycolysis model $\mathcal{M}^2_{glyco}$.

In the absence of allosteric regulation (model $\mathcal{M}^1_{neo}$), the flux $v_{46}(x^s_3, x^f_1, x^f_3, x^f_8)$ does not depend on the PEP concentration (x^f_8), that is, $\partial v_{46}/\partial x^f_8$ is set to 0. The other equations for model $\mathcal{M}^1_{glyco}$ are the same as for $\mathcal{M}^2_{glyco}$.

3 Derivation of interaction matrix for the reduced carbon assimilation model

We start from the reduced system of equations in Table 5, adapted for the glycolysis and gluconeogenesis cases as described in Sec. 2, and we apply the method presented in the main text. The aim is to infer the structure and the signs of the network of direct and indirect interactions between genes, for both the glycolysis and gluconeogenesis models. We remember from Sec. 2 in Text S2 that the main steps are

1. Compute the symbolic Jacobian M of the fast system and its inverse M^{-1}.
2. Compute the sign of the dependency of fast coupling variables (Crp·cAMP, free FruR, DNA supercoiling and RpoS·RssB complex) on the slow variables (enzymes, global regulators).
3. Compute the symbolic Jacobian $\mathcal{J}$ of the slow system.
4. Derive the interaction matrix between slow variables of the system, given by the sign of the elements of $\mathcal{J}$.

For both $\mathcal{M}_{glyco}$ and $\mathcal{M}_{neo}$, inspection of the stoichiometry matrix reveals the existence of three independent fast subsystems, each corresponding to a block in the stoichiometry matrix, describing changes in the DNA supercoiling level, RpoS degradation and glycolysis/gluconeogenesis, respectively. This allows step 1 to be performed independently for each

subsystem, thus simplifying the analysis and the computational costs of matrix inversion (Sec. 1 of Text S1). We notice here that the DNA supercoiling and RpoS modules do not depend on the specific growth conditions, so the computation is exactly the same in both the glycolytic and gluconeogenic case.

Most of the difficulties concern the third and largest module, which includes the enzymatic reactions involved in glycolysis/gluconeogenesis as well as cAMP synthesis and degradation, Crp·cAMP complex formation and the PTS signaling system. Indeed, 13 of the 17 fast variables of the network are included in this subsystem. In order to simplify the symbolic computation, we reduced the size of the Jacobian M by solving the algebraic equations for the complex formation reactions in the model. All symbolic computations, including matrix inversion, were performed using the Symbolic Math Toolbox (MathWorks). The MATLAB input files are available from the authors upon request.

3.1 Results for the gluconeogenesis model

In the Results section of the main text the interaction matrix of the glycolytic model $\mathcal{M}^2_{glyco}$ was shown. We report here the interaction matrix for growth on pyruvate, computed from $\mathcal{M}^2_{neo}$ (Table 6). We remind that genes *pykF* and*pfkA* do not appear as the corresponding enzymes were not included in the gluconeogenic model (Sec. 2.2). Non-regulated genes are also omitted from the matrix as their expression was assumed constant.

Bold signs indicate the dependencies that are due to allosteric effects and that are absent in the matrix $\mathcal{J}$ obtained from $\mathcal{M}^1_{neo}$. We stress that, unlike the glycolytic case, the gluconeogenesis network is completely sign-determined, both with and without allosteric regulation. Both for $\mathcal{M}^1_{neo}$ and $\mathcal{M}^2_{neo}$ conditions **C1-C4** are satisfied and the sign-determinedness is guaranteed. In particular, no antagonistic effects of Crp·cAMP and free FruR occur, as can be seen in Fig. 1*B* and *D* which compares the gene regulatory networks obtained from both models. During growth on pyruvate, in fact, the control of Crp·cAMP and free FruR concentrations does not involve the same glycolytic enzymes.

3.2 Results for the glycolysis model

In comparison with gluconeogenesis, the control of glycolysis is more complex. Networks for both $\mathcal{M}^1_{glyco}$ and $\mathcal{M}^2_{glyco}$ are denser than their counterparts for growth on pyruvate (Fig. 1).

In model $\mathcal{M}^1_{glyco}$, enzyme PykF is involved in the concentration control of both Crp·cAMP and free FruR, which have several common targets (*pgk*, *gapA* and *fbaA*). This is not allowed by condition **C4**. However, as the effect of PykF on the expression of the genes is the same, regardless of the intermediate fast coupling species, the sign-determinedness of the network is preserved. In particular, the concentration control coefficient of Crp·cAMP with respect to PykF is negative and Crp·cAMP activates the above-mentioned glycolytic genes, whereas the concentration control coefficient of free FruR with respect to PykF is positive and FruR is an inhibitor of the target genes.

In the presence of allosteric effects, the symbolic expressions obtained for the elements of $\mathcal{J}$ can be very complex and condition **C3** on the concentration control coefficients is not always satisfied. To illustrate this, we report two symbolic expressions, defining the effect during glycolysis of the Eno and PykF enzymes on the concentration of the metabolite FBP (and thus on free FruR). In order to facilitate the interpretation of the expressions below, we reformulate them by using the absolute value of negative partial derivatives.

$$
\begin{aligned}
\frac{\partial x^f_3}{\partial x^s_9} &= -\frac{\partial v_{52}}{\partial x^s_9}\Big|\frac{\partial v_{51}}{\partial x^f_7}\Big|\Big|\frac{\partial v_{50}}{\partial x^f_6}\Big|\Big|\frac{\partial v_{49}}{\partial x^f_5}\Big|\Big|\frac{\partial v_{47}}{\partial x^f_4}\Big|(82\Big|\frac{\partial v_{55}}{\partial x^f_9}\Big|\frac{\partial v_{53}}{\partial x^f_8}\frac{\partial v_{65}}{\partial x^f_{10}}+ \\
&\quad ((45\frac{\partial v_{53}}{\partial x^f_8}-37\frac{\partial v_{55}}{\partial x^f_8})\frac{\partial v_{65}}{\partial x^f_{10}}+45\frac{\partial v_{55}}{\partial x^f_{10}}\frac{\partial v_{53}}{\partial x^f_8})\frac{\partial v_{63}}{\partial x^f_9})/D
\end{aligned}
\tag{1}
$$

$$
\begin{aligned}
\frac{\partial x^f_3}{\partial x^s_{10}} &= -\frac{\partial v_{53}}{\partial x^s_{10}}(82\Big|\frac{\partial v_{55}}{\partial x^f_9}\Big|\frac{\partial v_{52}}{\partial x^f_8}\Big|\frac{\partial v_{51}}{\partial x^f_7}\Big|\frac{\partial v_{50}}{\partial x^f_6}\Big|\frac{\partial v_{49}}{\partial x^f_5}\Big|\Big|\frac{\partial v_{47}}{\partial x^f_4}\Big|\frac{\partial v_{65}}{\partial x^f_{10}}+((((((82\frac{\partial v_{55}}{\partial x^f_8} \\
&\quad +45\Big|\frac{\partial v_{52}}{\partial x^f_8}\Big|)\Big|\frac{\partial v_{51}}{\partial x^f_7}\Big|+82\frac{\partial v_{52}}{\partial x^f_7}\frac{\partial v_{55}}{\partial x^f_8})\Big|\frac{\partial v_{50}}{\partial x^f_6}\Big|+82\frac{\partial v_{51}}{\partial x^f_6}\frac{\partial v_{52}}{\partial x^f_7}\frac{\partial v_{55}}{\partial x^f_8})\Big|\frac{\partial v_{49}}{\partial x^f_5}\Big| \\
&\quad +82\frac{\partial v_{50}}{\partial x^f_5}\frac{\partial v_{51}}{\partial x^f_6}\frac{\partial v_{52}}{\partial x^f_7}\frac{\partial v_{55}}{\partial x^f_8})\Big|\frac{\partial v_{47}}{\partial x^f_4}\Big|+40\frac{\partial v_{49}}{\partial x^f_4}\frac{\partial v_{50}}{\partial x^f_5}\frac{\partial v_{51}}{\partial x^f_6}\frac{\partial v_{52}}{\partial x^f_7}\frac{\partial v_{55}}{\partial x^f_8})\frac{\partial v_{65}}{\partial x^f_{10}} \\
&\quad +45\Big|\frac{\partial v_{55}}{\partial x^f_{10}}\Big|\Big|\frac{\partial v_{52}}{\partial x^f_8}\Big|\Big|\frac{\partial v_{51}}{\partial x^f_7}\Big|\Big|\frac{\partial v_{50}}{\partial x^f_6}\Big|\Big|\frac{\partial v_{49}}{\partial x^f_5}\Big|\Big|\frac{\partial v_{47}}{\partial x^f_4}\Big|)\frac{\partial v_{63}}{\partial x^f_9})/D
\end{aligned}
\tag{2}
$$

where D is the determinant of the fast system:

$$
\begin{aligned}
D &= 45\frac{\partial v_{49}}{\partial x^f_4}\frac{\partial v_{50}}{\partial x^f_5}\frac{\partial v_{51}}{\partial x^f_6}\frac{\partial v_{52}}{\partial x^f_7}\frac{\partial v_{63}}{\partial x^f_9}\frac{\partial v_{65}}{\partial x^f_{10}}\frac{\partial v_{47}}{\partial x^f_3}\frac{\partial v_{53}}{\partial x^f_8}+82\frac{\partial v_{52}}{\partial x^f_7}\frac{\partial v_{53}}{\partial x^f_3}\frac{\partial v_{55}}{\partial x^f_8}\frac{\partial v_{63}}{\partial x^f_9}\Big|\frac{\partial v_{50}}{\partial x^f_6}\Big|\frac{\partial v_{49}}{\partial x^f_5}\frac{\partial v_{47}}{\partial x^f_4}\frac{\partial v_{65}}{\partial x^f_{10}}+ \\
&\quad 82\frac{\partial v_{49}}{\partial x^f_4}\frac{\partial v_{50}}{\partial x^f_5}\frac{\partial v_{51}}{\partial x^f_6}\frac{\partial v_{52}}{\partial x^f_7}\frac{\partial v_{55}}{\partial x^f_9}\frac{\partial v_{47}}{\partial x^f_3}\frac{\partial v_{53}}{\partial x^f_8}\frac{\partial v_{65}}{\partial x^f_{10}}+82\frac{\partial v_{50}}{\partial x^f_5}\frac{\partial v_{51}}{\partial x^f_6}\frac{\partial v_{52}}{\partial x^f_7}\frac{\partial v_{53}}{\partial x^f_3}\frac{\partial v_{55}}{\partial x^f_8}\frac{\partial v_{63}}{\partial x^f_9}\frac{\partial v_{47}}{\partial x^f_4}\frac{\partial v_{65}}{\partial x^f_{10}}+ \\
&\quad 82\frac{\partial v_{53}}{\partial x^f_3}\frac{\partial v_{55}}{\partial x^f_9}\frac{\partial v_{52}}{\partial x^f_8}\frac{\partial v_{51}}{\partial x^f_7}\Big|\frac{\partial v_{50}}{\partial x^f_6}\Big|\frac{\partial v_{49}}{\partial x^f_5}\frac{\partial v_{47}}{\partial x^f_4}\frac{\partial v_{65}}{\partial x^f_{10}}+82\frac{\partial v_{51}}{\partial x^f_6}\frac{\partial v_{52}}{\partial x^f_7}\frac{\partial v_{53}}{\partial x^f_3}\frac{\partial v_{55}}{\partial x^f_8}\frac{\partial v_{63}}{\partial x^f_9}\frac{\partial v_{49}}{\partial x^f_5}\frac{\partial v_{47}}{\partial x^f_4}\frac{\partial v_{65}}{\partial x^f_{10}}+ \\
&\quad 40\frac{\partial v_{49}}{\partial x^f_4}\frac{\partial v_{50}}{\partial x^f_5}\frac{\partial v_{51}}{\partial x^f_6}\frac{\partial v_{52}}{\partial x^f_7}\frac{\partial v_{53}}{\partial x^f_3}\frac{\partial v_{55}}{\partial x^f_8}\frac{\partial v_{63}}{\partial x^f_9}\frac{\partial v_{65}}{\partial x^f_{10}}+45\frac{\partial v_{53}}{\partial x^f_3}\frac{\partial v_{63}}{\partial x^f_9}\Big|\frac{\partial v_{55}}{\partial x^f_{10}}\Big|\frac{\partial v_{52}}{\partial x^f_8}\frac{\partial v_{51}}{\partial x^f_7}\Big|\frac{\partial v_{50}}{\partial x^f_6}\Big|\frac{\partial v_{49}}{\partial x^f_5}\frac{\partial v_{47}}{\partial x^f_4}- \\
&\quad 37\frac{\partial v_{49}}{\partial x^f_4}\frac{\partial v_{50}}{\partial x^f_5}\frac{\partial v_{51}}{\partial x^f_6}\frac{\partial v_{52}}{\partial x^f_7}\frac{\partial v_{55}}{\partial x^f_8}\frac{\partial v_{63}}{\partial x^f_9}\frac{\partial v_{65}}{\partial x^f_{10}}\frac{\partial v_{47}}{\partial x^f_3}+45\frac{\partial v_{53}}{\partial x^f_3}\frac{\partial v_{63}}{\partial x^f_9}\frac{\partial v_{65}}{\partial x^f_{10}}\frac{\partial v_{52}}{\partial x^f_8}\frac{\partial v_{51}}{\partial x^f_7}\Big|\frac{\partial v_{50}}{\partial x^f_6}\Big|\frac{\partial v_{49}}{\partial x^f_5}\frac{\partial v_{47}}{\partial x^f_4}+ \\
&\quad 45\frac{\partial v_{49}}{\partial x^f_4}\frac{\partial v_{50}}{\partial x^f_5}\frac{\partial v_{51}}{\partial x^f_6}\frac{\partial v_{52}}{\partial x^f_7}\frac{\partial v_{63}}{\partial x^f_9}\Big|\frac{\partial v_{55}}{\partial x^f_{10}}\Big|\frac{\partial v_{47}}{\partial x^f_3}\frac{\partial v_{53}}{\partial x^f_8}+82\frac{\partial v_{53}}{\partial x^f_3}\frac{\partial v_{55}}{\partial x^f_8}\frac{\partial v_{63}}{\partial x^f_9}\frac{\partial v_{51}}{\partial x^f_7}\Big|\frac{\partial v_{50}}{\partial x^f_6}\Big|\frac{\partial v_{49}}{\partial x^f_5}\frac{\partial v_{47}}{\partial x^f_4}\frac{\partial v_{65}}{\partial x^f_{10}}.
\end{aligned}
\tag{3}
$$

The stability condition for the fast system imposes $(-1)^{n-m}D > 0$, where $n-m$ is the system dimension (see the Methods section of the main text). In our case, given the even number of fast equations in the system, D is thus positive. As the expression in the numerator is also positive, this information is sufficient to infer that the sign of the concentration control coefficient $\partial x^f_3/\partial x^s_{10}$ is negative, that is, the FBP concentration decreases in response to an increase in the PykF concentration.

In contrast, the control coefficient of FBP with respect to Eno, $\partial x^f_3/\partial x^s_9$, involves a difference between positive terms and therefore gives rise to an ambiguity. The computation of the dependency of FBP on FbaA, GapA, and Pgk also leads to an ambiguity, caused by the same algebraic subexpression as that occurring in the numerator of $\partial x^f_3/\partial x^s_9$. This means that all undefined cases can be simultaneously solved by an experimental measurement of FBP dependence on either Eno, FbaA, GapA, or Pgk. As reported in the main text, we used data from [11] showing that an overexpression of FbaA decreases FBP levels. This allowed us to satisfy condition **C3**, defining the sign of all free FruR concentration control coefficients.

On the other hand, condition **C4** cannot be satisfied. In model $\mathcal{M}^2_{glyco}$, in fact, almost all enzymes participate in both the control of Crp·cAMP and free FruR. This gives rise to a competitive control of genes *pgk*, *gapA*, and *fbaA* by enzymes Eno, Pgk, GapA, and FbaA that cannot be resolved without additional quantitative information on gene expression patterns.

4 Feedback loops in carbon assimilation network

From Eq. 6 in the main text we recover a matrix $G = \text{sign}(\mathcal{J}) \in \{-1, 0, +1\}$ containing the signs of the elements of the Jacobian matrix. The complexity of the emerging network can be characterized by its connectivity and by the number and length of the feedback loops.

Formally, a *feedback loop* is defined as a non-empty sequence $\mathcal{L}$ of matrix elements, say

$$g_{i_1 i_2}\, g_{i_2 i_3} \cdots g_{i_{l-1} i_l}, \tag{4}$$

such that (i) $i_l = i_1$ (the sequence is a circuit) and (ii) $i_j \neq i_k$ for all $j, k \in]1, l[$ (the circuit is elementary) [13, 14]. A feedback loop is said to be positive if the product of the signs of the elements in Eq. 4 is positive. Otherwise, it is said to be negative. The maximal loop length is defined as $\max_{\mathcal{L}} l$, for all $\mathcal{L} \in G$. Enumeration of the feedback loops is performed by a simple recursive algorithm that, starting from each possible index i, visits all connected elements (*i.e.*, $g_{ik} \neq 0$), and checks for circuits.

The connectivity of a gene i is defined as the cardinality of the set $\{k \mid g_{ik} \neq 0\}$, *i.e.* the number of its regulating proteins. At the network level we simply consider the average connectivity per gene.

The above measures are used in the main text to compare the networks obtained from the glycolysis and gluconeogenesis models. To put the analysis in perspective we defined a reference model of the network, $\mathcal{M}^0$, as including only direct interactions on the transcriptional level. This model thus account for purely transcriptional dependencies between genes, which are not mediated by metabolism. Strictly speaking, the direct interactions are given by the first term in the right-hand side of Eq. 6 in the main text, *e.g.*, the regulation of *topA* by Fis. We relax the definition somewhat by including the regulation exerted by transcription factors modified by metabolic effectors, such as Crp, which are formally included in the second term of Eq. 6. This allows our results to be compared with those obtained in previous studies.

References

1. Heinrich, R. and Schuster, S. (1996) *The Regulation of Cellular Systems.* Chapman & Hall, New York.

2. Reder, C. Metabolic control theory: a structural approach. *J. Theor. Biol.*, **135**, 175–201, 1988

3. Bettenbrock, K. et al. A quantitative approach to catabolite repression in *Escherichia coli. J. Biol. Chem.*, **281**, 2578–2584, 2005

4. Kremling, A., Bettenbrock, K., and Gilles, E. Analysis of global control of *Escherichia coli* carbohydrate uptake. *BMC Syst. Biol.*, **1**, 42, 2007

5. Chassagnole, C., Noisommit-Rizzi, N., Schmid, J., Mauch, K., and Reuss, M. Dynamic modeling of the central carbon metabolism of *Escherichia coli. Biotechnol. Bioeng.*, **79**, 53–73, 1988

6. Ropers, D.,de Jong, H., Page M., Schneider D., Geiselmann J. Qualitative simulation of the carbon starvation response in *Escherichia coli. Biosystems*, **84**, 124–152, 2006

7. Ropers, D., Baldazzi, V. & de Jong, H. , Model reduction using piecewise-linear approximations preserves dynamic properties of the carbon starvation response in *Escherichia coli*, *ACM/IEEE Trans. Comput. Biol. Bioinf.*, 2009 In press.

8. Nicolas C., *et al.* Response of the central metabolism of *Escherichia coli* to modified expression of the gene encoding the glucose-6-phosphate dehydrogenase. *FEBS Lett.*, 581(20):3771–3776, 2007.

9. Keseler, Ingrid M. *et al.* (2009) EcoCyc: A comprehensive view of *Escherichia coli* biology *Nucl. Acids Res*, **37** , D464-470

10. Saier, M. J., Ramseier, T., and J., R. Regulation of carbon utilization. In Neidhardt, F., Curtiss III, R., Ingraham, J., Lin, E., Low, K., Magasanik, B., Reznikoff, W., Riley, M., Schaechter, M., and Umbarger, H. (eds.), *Escherichia coli and Salmonella: Cellular and Molecular Biology*, pp. 1325–43. ASM Press, Washington D.C, 1996 .

11. Babul, J., Clifton, D., Kretschmer, M., and Fraenkel, D. G. Glucose metabolism in *Escherichia coli* and the effect of increased amount of aldolase. *Biochemistry*, **32**, 4685–4692, 1993

12. Bettenbrock, K. and Sauter, T. and Jahreis, K. and Kremling, A. and Lengeler, J.W. and Gilles, E.-D. Correlation between growth rates, EIIACrr phosphorylation, and intracellular cyclic AMP levels in *Escherichia coli* K-12 *J. Bacteriol.*, **189**, 6891–6900, 2007

13. Richard, A. and Comet, J.-P. Necessary conditions for multistationarity in discrete dynamical systems. *Discr. Appl. Math.*, **155**, 2403 – 2413, 2007.

14. Soulé, C. Mathematical approaches to differentiation and gene regulation. *C. R. Biol.*, **329**, 13–20, 2006.

15. Ricci J.C.D. Influence of phosphoenolpyruvate on the dynamic behavior of phosphofructokinase of *Escherichia coli. J. Theor. Biol.*, 178(2):145–150, 1996.

16. Richter O., Betz A., and Giersch C. The response of oscillating glycolysis in the NADH/NAD system: A comparison between experiment and a computer model. *BioSystems*, 7:137–146, 1975.

17. Hellinga H. W.and Evans P. R. Nucleotide sequence and high-level expression of the major *Escherichia coli* phosphofructokinase. *Eur. J. Biochem.*, 149(2):363–373, 1985.

18. Nègre D., *et al.* Definition of a consensus DNA-binding site for the *Escherichia coli* pleiotropic regulatory protein, FruR. *Mol. Microbiol.*, 21(2):257–266, 1996.

19. Bardey V., *et al.* Characterization of the molecular mechanisms involved in the differential production of erythrose-4-phosphate dehydrogenase, 3-phosphoglycerate kinase and class II fructose-1,6-bisphosphate aldolase in *Escherichia coli. Mol. Microbiol.*, 57(5):1265–1287, 2005.

20. Charpentier B., Bardey V., Robas N., and Branlant C. The EIIGlc protein is involved in glucose-mediated activation of *Escherichia coli gapA* and *gapB-pgk* transcription. *J. Bacteriol.*, 180:6476–6483, 1998.

21. Ramseier T. M. , Bledig S., Michotey V., Feghali R., and Saier M. H. The global regulatory protein FruR modulates the direction of carbon flow in *Escherichia coli*. *Mol. Microbiol.*, 16(6):1157–1169, 1995.

22. Charpentier B.and Branlant C.. The *Escherichia coli gapA* gene is transcribed by the vegetative RNA polymerase holoenzyme E sigma 70 and by the heat shock RNA polymerase E sigma 32. *J. Bacteriol.*, 176(3):830–839, 1994.

23. Shimada T., Fujita N., Maeda M., and Ishihama A. Systematic search for the Cra-binding promoters using genomic SELEX system. *Genes Cells*, 10(9):907–918, 2005.

24. Weng M., Makaroff C. A., and Zalkin H. Nucleotide sequence of *Escherichia coli pyrG* encoding CTP synthetase. *J. Biol. Chem.*, 261(12):5568–5574, 1986.

25. Bledig S. A., Ramseier T. M., and Saier M. H. FruR mediates catabolite activation of pyruvate kinase (*pykF)* gene expression in *Escherichia coli*. *J Bacteriol*, 178:280–283, 1996.

26. Sauter T. and Gilles E. D. Modeling and experimental validation of the signal transduction via the *Escherichia coli* sucrose phospho transferase system. *J Biotechnol*, 110(2):181–199, 2004.

27. Geerse R. H., van der Pluijm J., and Postma P. W. The repressor of the PEP:fructose phosphotransferase system is required for the transcription of the *pps* gene of *Escherichia coli*. *Mol. Gen. Genet.*, 218:348–352, 1989.

28. Nègre D., *et al.* FruR-mediated transcriptional activation at the *ppsA* promoter of *Escherichia coli*. *J. Mol. Biol.*, 276:355–365, 1998.

29. Aiba H. Transcription of the *Escherichia coli* adenylate cyclase gene is negatively regulated by cAMP-cAMP receptor protein. *J. Biol. Chem.*, 260(5):3063–70, 1985.

30. Inada T., Takahashi H., Mizuno T., and Aiba H.. Down regulation of cAMP production by cAMP receptor protein in *Escherichia coli*: an assessment of the contributions of transcriptional and posttranscriptional control of adenylate cyclase. *Mol. Gen. Genet.*, 253(1-2):198–204, 1996.

31. Kawamukai M., Kishimoto J.,Utsumi R., Himeno M., Komano T., and Aiba H. Negative regulation of adenylate cyclase gene (*cya*) expression by cyclic AMP-cyclic AMP receptor protein in *Escherichia coli*: studies with *cya*-lac protein and operon fusion plasmids. *J. Bacteriol.*, 164(2):872–7, 1985.

32. Ishizuka H., Hanamura A., Inada T., and Aiba H. Mechanism of the down-regulation of cAMP receptor protein by glucose in *Escherichia coli*: role of autoregulation of the *crp* gene. *EMBO J.*, 13(13):3077–82, 1994.

33. Gonzalez-Gil G., Kahmann R., and Muskhelishvili G. Regulation of *crp* transcription by oscillation between distinct nucleoprotein complexes. *EMBO J.*, 17(10):2877–85, 1998.

34. Nasser W., Schneider R., Travers A. , and Muskhelishvili G. CRP modulates *fis* transcription by alternate formation of activating and repressing nucleoprotein complexes. *J. Biol. Chem.*, 276 (21): 17878–86, 2001.

35. Ninnemann O., Koch C., and Kahmann R. The *E. coli fis* promoter is subject to stringent control and autoregulation. *EMBO J.*, 11 (3): 1075–83, 1992.

36. Schneider R., Travers A., and Muskhelishvili G. The expression of the *Escherichia coli fis* gene is strongly dependent on the superhelical density of DNA. *Mol. Microbiol.*, 38 (1): 165–75, 2000.

37. Menzel R. and Gellert M. Regulation of the genes for *E. coli* DNA gyrase: homeostatic control of DNA supercoiling. *Cell*, 34(1):105–13, 1983.

38. Menzel R. and Gellert M. Modulation of transcription by DNA supercoiling: a deletion analysis of the *Escherichia coli gyrA* and *gyrB* promoters. *Proc. Natl. Acad. Sci. USA*, 84(12):4185–9, 1987.

39. Baquero M. R., Bouzon M., Varea J., and Moreno F. *sbmC*, a stationary-phase induced SOS *Escherichia coli* gene, whose product protects cells from the DNA replication inhibitor microcin B17. *Mol. Microbiol.*, 18(02):301–311, 1995.

40. Zheng D., Constantinidou C., Hobman J.L., and Minchin S.D. Identification of the CRP regulon using *in vitro* and *in vivo* transcriptional profiling. *Nucleic Acids Res.*, 32(19):5874–5893, 2004.

41. Oh T.J. , Jung I.L., and Kim I.G. The *Escherichia coli* SOS gene *sbmC* is regulated by H-NS and RpoS during the SOS induction and stationary growth phase. *Biochem. Biophys. Res. Commun.*, 288(4):1052–8, 2001.

42. Weinstein-Fischer D., Elgrably-Weiss M., and Altuvia S. *Escherichia coli* response to hydrogen peroxide: a role for DNA supercoiling, topoisomerase I and Fis. *Mol. Microbiol.*, 35(6):1413–20, 2000.

43. Tse-Dinh Y.C. Regulation of the *Escherichia coli* DNA topoisomerase I gene by DNA supercoiling. *Nucleic Acids Res*, 13(13):4751–63, 1985.

44. Tse-Dinh Y.C. and Beran R.K. Multiple promoters for transcription of the *Escherichia coli* DNA topoisomerase I gene and their regulation by DNA supercoiling. *J. Mol. Biol.*, 202(4):735–42, 1988.

45. Becker G., Klauck E. , and Hengge-Aronis R. The response regulator RssB, a recognition factor for σ^S proteolysis in *Escherichia coli*, can act like an anti-σ^S factor. *Mol. Microbiol.*, 35(3):657–666, 2000.

46. Pruteanu M. and Hengge-Aronis R. The cellular level of the recognition factor RssB is rate-limiting for σ^s proteolysis: implications for RssB regulation and signal transduction in σ^s turnover in *Escherichia coli*. *Mol. Microbiol.*, 45(6):1701–1714, 2002.

47. Peterson C.N., Ruiz N., and Silhavy T.J. RpoS proteolysis is regulated by a mechanism that does not require the SprE (RssB) response regulator phosphorylation site. *J. Bacteriol.*, 186(21):7403–7410, 2004.

48. Ruiz N., Peterson C.N., and Silhavy T.J. RpoS-dependent transcriptional control of *sprE*: Regulatory feedback loop. *J. Bacteriol.*, 183 (20): 5974–5981, 2001.

49. Murray H.D. and Gourse R.L. Unique roles of the *rrn* P2 rRNA promoters in *Escherichia coli*. *Mol. Microbiol.*, 52 (5): 1375–87, 2004.

50. Schneider D.A. , Ross W., and Gourse R.L. Control of rRNA expression in *Escherichia coli*. *Curr. Opin. Microbiol.*, 6(2): 151–6, 2003.

51. Ramseier T. M. , *et al.* In vitro binding of the pleiotropic transcriptional regulatory protein, FruR, to the *fru*, *pps*, *ace*, *pts* and *icd* operons of *Escherichia coli* and *Salmonella Typhimurium*. *J Mol Biol*, 234:28–44, 1993.

52. Ball C.A., Osuna R.,. Ferguson K.C, and Johnson R.C. Dramatic changes in Fis levels upon nutrient upshift in *Escherichia coli*. *J. Bacteriol.*, 174(24):8043–56, 1992.

53. Mandel M.J. and Silhavy T.J. Starvation for different nutrients in *Escherichia coli* results in differential modulation of RpoS levels and stability. *J. Bacteriol.*, 187(2):434–442, 2005.

54. Zgurskaya H.I. , Keyhan M., and Matin A. The σ^s level in starving *Escherichia coli* cells increases solely as a result of its increased stability, despite decreased synthesis. *Mol. Microbiol.*, 24(3):643–652, 1997.

55. Fraser A.D. and Yamazaki H. Effect of carbon sources on the rates of cyclic AMP synthesis, excretion, and degradation, and the ability to produce beta-galactosidase in *Escherichia coli*. *Can. J. Biochem.*, 57(8):1073–9, 1979.

56. Yang J.K. and Epstein W. Purification and characterization of adenylate cyclase from *Escherichia coli* K12. *J. Biol. Chem.*, 258(6):3750–8, 1983.

57. Chatterji M. and Nagaraja V. GyrI: a counter-defensive strategy against proteinaceous inhibitors of DNA gyrase. *EMBO Rep*, 3(3):261–7, 2002.

58. Bledig, S., Ramseier, T., and Saier, M.H (1996) FruR mediates catabolite activation of pyruvate kinase (*pykF*) gene expression in *Escherichia coli*. *J. Bacteriol.*, **178**, 280–283.

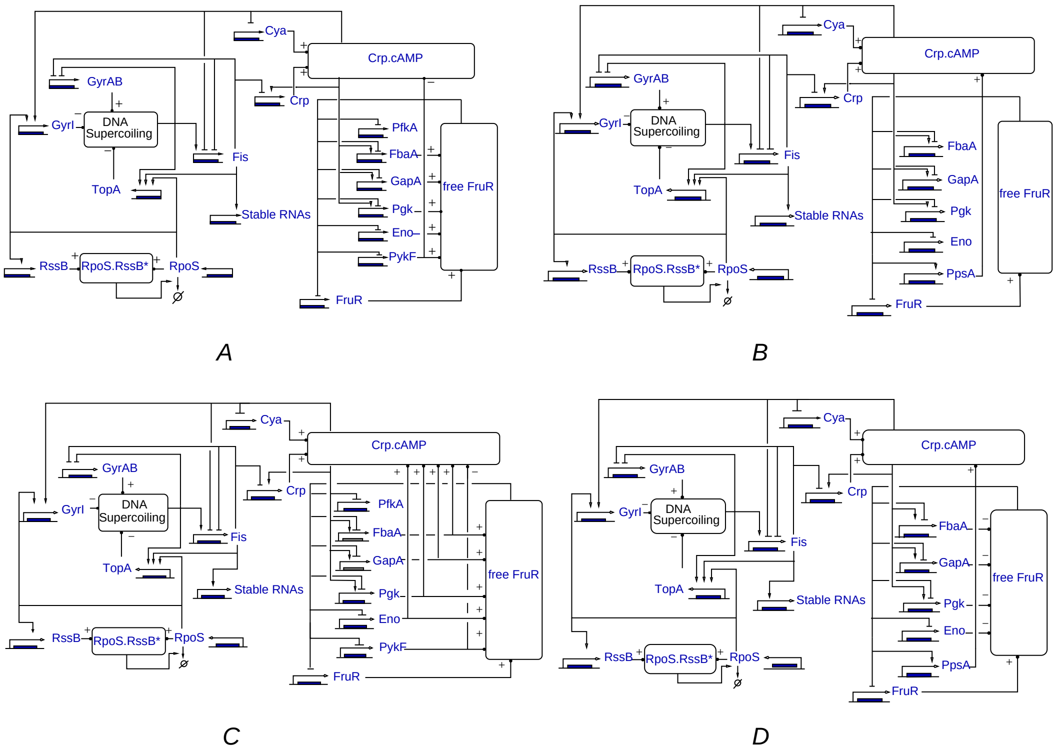

Figure 1: **Reconstructed networks of direct and indirect interactions for all considered models.** *A*: Network for model $\mathcal{M}^1_{glyco}$, during glycolysis and without the inclusion of allosteric regulation. *B*: Network for model $\mathcal{M}^1_{neo}$, during gluconeogenesis and in the absence of allosteric regulation. *C*: Network for model $\mathcal{M}^2_{glyco}$ for glycolysis. Allosteric regulation gives rise to additional control of the Crp·cAMP concentration by glycolytic enzymes. *D*: Network for model $\mathcal{M}^2_{neo}$ for gluconeogenesis. In this case, the presence of allosteric regulation is responsible for the control of free FruR concentration by glycolytic enzymes.

Name	Variable	Name	Variable
Pgi	x_1	Pfka	x_2
Fbp	x_3	FbaA	x_4
TpiA	x_5	GapA	x_6
Pgk	x_7	GpmI	x_8
Eno	x_9	PykF	x_{10}
PpsA	x_{11}	Cya	x_{12}
free Crp	x_{13}	Fis	x_{14}
free GyrAB	x_{15}	free GyrI	x_{16}
TopA	x_{17}	free RpoS	x_{18}
free RssB	x_{19}	Stable RNAs	x_{20}
free FruR	x_{21}	G6P	x_{22}
F6P	x_{23}	FBP	x_{24}
DHAP	x_{25}	G3P	x_{26}
DPG	x_{27}	3PG	x_{28}
2PG	x_{29}	PEP	x_{30}
Pyr	x_{31}	PTS	x_{32}
cAMP	x_{33}	PTSp	x_{34}
Crp·cAMP	x_{35}	FruR·FBP	x_{36}
GyrAB·GyrI	x_{37}	free RssB*	x_{38}
RpoS·RssB*	x_{39}	DNA supercoiling	x_{40}

Table 1: Table of variables for the complete carbon assimilation model.

Protein synthesis					
v_1				Pgi	[9]
$v_2(x_{21})$	$\frac{\partial v_2}{\partial x_{21}} < 0$			PfkA	[17, 18]
v_3				Fbp	[9]
$v_4(x_{21}, x_{35})$	$\frac{\partial v_4}{\partial x_{21}} < 0$	$\frac{\partial v_4}{\partial x_{35}} > 0$		FbaA	[19, 20, 21]
v_5				TpiA	[9]
$v_6(x_{21}, x_{35})$	$\frac{\partial v_6}{\partial x_{21}} < 0$	$\frac{\partial v_6}{\partial x_{35}} > 0$		GapA	[22, 23]
$v_7(x_{21}, x_{35})$	$\frac{\partial v_7}{\partial x_{21}} < 0$	$\frac{\partial v_7}{\partial x_{35}} > 0$		Pgk	[19, 20, 21]
v_8				GpmI	[9]
$v_9(x_{21})$	$\frac{\partial v_9}{\partial x_{21}} < 0$			Eno	[23, 24]
$v_{10}(x_{21})$	$\frac{\partial v_{10}}{\partial x_{21}} < 0$			PykF	[58]
$v_{11}(x_{21})$	$\frac{\partial v_{11}}{\partial x_{21}} > 0$			PpsA	[27, 28]
$v_{12}(x_{35})$	$\frac{\partial v_{12}}{\partial x_{35}} < 0$			Cya	[29, 30, 31]
$v_{13}(x_{14}, x_{35})$	$\frac{\partial v_{13}}{\partial x_{14}} < 0$	$\frac{\partial v_{13}}{\partial x_{35}} > 0$		Crp	[33, 32]
$v_{14}(x_{14}, x_{35}, x_{40})$	$\frac{\partial v_{14}}{\partial x_{14}} < 0$	$\frac{\partial v_{14}}{\partial x_{35}} < 0$	$\frac{\partial v_{14}}{\partial x_{40}} > 0$	Fis	[34, 35, 36]
$v_{15}(x_{14}, x_{40})$	$\frac{\partial v_{15}}{\partial x_{14}} < 0$	$\frac{\partial v_{15}}{\partial x_{40}} < 0$		GyrAB	[37, 38]
$v_{16}(x_{18}, x_{35})$	$\frac{\partial v_{16}}{\partial x_{18}} > 0$	$\frac{\partial v_{16}}{\partial x_{35}} > 0$		GyrI	[39, 40, 41]
$v_{17}(x_{14}, x_{18}, x_{40})$	$\frac{\partial v_{17}}{\partial x_{14}} > 0$	$\frac{\partial v_{17}}{\partial x_{18}} > 0$	$\frac{\partial v_{17}}{\partial x_{40}} > 0$	TopA	[42, 43, 44]
v_{18}				RpoS	[9]
$v_{19}(x_{18})$	$\frac{\partial v_{19}}{\partial x_{18}} > 0$			RssB	[46, 48]
$v_{20}(x_{14})$	$\frac{\partial v_{20}}{\partial x_{14}} > 0$			rrn	[49, 50]
$v_{21}(x_{21})$	$\frac{\partial v_{21}}{\partial x_{21}} < 0$			FruR	[51]
Protein degradation					
$v_{22}(x_1)$	$\frac{\partial v_{22}}{\partial x_1} > 0$			Pgi	
$v_{23}(x_2)$	$\frac{\partial v_{23}}{\partial x_2} > 0$			PfkA	
$v_{24}(x_3)$	$\frac{\partial v_{24}}{\partial x_3} > 0$			Fbp	
$v_{25}(x_4)$	$\frac{\partial v_{25}}{\partial x_4} > 0$			FbaA	
$v_{26}(x_5)$	$\frac{\partial v_{26}}{\partial x_5} > 0$			TpiA	
$v_{27}(x_6)$	$\frac{\partial v_{27}}{\partial x_6} > 0$			GapA	
$v_{28}(x_7)$	$\frac{\partial v_{28}}{\partial x_7} > 0$			Pgk	
$v_{29}(x_8)$	$\frac{\partial v_{29}}{\partial x_8} > 0$			GpmI	
$v_{30}(x_9)$	$\frac{\partial v_{30}}{\partial x_9} > 0$			Eno	
$v_{31}(x_{10})$	$\frac{\partial v_{31}}{\partial x_{10}} > 0$			PykF	
$v_{32}(x_{11})$	$\frac{\partial v_{32}}{\partial x_{11}} > 0$			PpsA	
$v_{33}(x_{12})$	$\frac{\partial v_{33}}{\partial x_{12}} > 0$			Cya	
$v_{34}(x_{13/35})$	$\frac{\partial v_{34}}{\partial x_{13}} > 0$			Crp	
$v_{35}(x_{14})$	$\frac{\partial v_{35}}{\partial x_{14}} > 0$			Fis	[52]
$v_{36}(x_{15/37})$	$\frac{\partial v_{36}}{\partial x_{15}} > 0$			GyrAB	
$v_{37}(x_{16})$	$\frac{\partial v_{37}}{\partial x_{16}} > 0$			GyrI	
$v_{38}(x_{17})$	$\frac{\partial v_{38}}{\partial x_{17}} > 0$			TopA	
$v_{39}(x_{18/39})$	$\frac{\partial v_{39}}{\partial x_{18}} > 0$			RpoS	[53, 54]
$v_{40}(x_{39})$	$\frac{\partial v_{40}}{\partial x_{39}} > 0$			RpoS·RssB*	
$v_{41}(x_{19/38})$	$\frac{\partial v_{41}}{\partial x_{19}} > 0$			RssB	
$v_{42}(x_{20})$	$\frac{\partial v_{42}}{\partial x_{20}} > 0$			rrn	
$v_{43}(x_{21/36})$	$\frac{\partial v_{43}}{\partial x_{21}} > 0$			FruR	

Table 2: Table of reaction rates for the complete carbon assimilation model, with references to the literature for supporting evidence.

Enzymatic and signaling reactions						
$v_{44}(x_1, x_{22}, x_{23})$	$\frac{\partial v_{44}}{\partial x_1} \leqslant 0$	$\frac{\partial v_{44}}{\partial x_{22}} > 0$	$\frac{\partial v_{44}}{\partial x_{23}} < 0$		Phosphoglucose isomerase (Pgi)	[3]
$v_{45}(x_2, x_{23}, x_{30})$	$\frac{\partial v_{45}}{\partial x_2} \leqslant 0$	$\frac{\partial v_{45}}{\partial x_{23}} > 0$	$\frac{\partial v_{45}}{\partial x_{30}} < 0$		Phosphofructokinase (PfkA)	[3, 15]
$v_{46}(x_3, x_{22}, x_{24}, x_{30})$	$\frac{\partial v_{46}}{\partial x_3} \leqslant 0$	$\frac{\partial v_{46}}{\partial x_{22}} > 0$	$\frac{\partial v_{46}}{\partial x_{24}} < 0$	$\frac{\partial v_{46}}{\partial x_{30}} < 0$	Fructose-biphosphatase (Fbp)	[3]
$v_{47}(x_4, x_{24}, x_{25}, x_{26})$	$\frac{\partial v_{47}}{\partial x_4} \leqslant 0$	$\frac{\partial v_{47}}{\partial x_{24}} > 0$	$\frac{\partial v_{47}}{\partial x_{25}} < 0$		Fructose-biphosphate aldolase (FbaA)	[16]
$v_{48}(x_5, x_{25}, x_{26})$	$\frac{\partial v_{48}}{\partial x_5} \leqslant 0$	$\frac{\partial v_{48}}{\partial x_{25}} > 0$	$\frac{\partial v_{48}}{\partial x_{26}} < 0$		Triose-phosphate isomerase (TpiA)	
$v_{49}(x_6, x_{26}, x_{27})$	$\frac{\partial v_{49}}{\partial x_6} \leqslant 0$	$\frac{\partial v_{49}}{\partial x_{26}} > 0$	$\frac{\partial v_{49}}{\partial x_{27}} < 0$		Glyceraldehyde-3-phosphate dehydrogenase (GapA)	[3]
$v_{50}(x_7, x_{27}, x_{28})$	$\frac{\partial v_{50}}{\partial x_7} \leqslant 0$	$\frac{\partial v_{50}}{\partial x_{27}} > 0$	$\frac{\partial v_{50}}{\partial x_{28}} < 0$		Phosphoglycerate kinase (Pgk)	
$v_{51}(x_8, x_{28}, x_{29})$	$\frac{\partial v_{51}}{\partial x_8} \leqslant 0$	$\frac{\partial v_{51}}{\partial x_{28}} > 0$	$\frac{\partial v_{51}}{\partial x_{29}} < 0$		Phosphoglycerate mutase (GpmI)	
$v_{52}(x_9, x_{29}, x_{30})$	$\frac{\partial v_{52}}{\partial x_9} \leqslant 0$	$\frac{\partial v_{52}}{\partial x_{29}} > 0$	$\frac{\partial v_{52}}{\partial x_{30}} < 0$		Enolase (Eno)	[3]
$v_{53}(x_{10}, x_{30}, x_{24})$	$\frac{\partial v_{53}}{\partial x_{10}} \leqslant 0$	$\frac{\partial v_{53}}{\partial x_{30}} > 0$	$\frac{\partial v_{53}}{\partial x_{24}} > 0$		Pyruvate kinase (PykF)	[3, 26]
$v_{54}(x_{11}, x_{30}, x_{31})$	$\frac{\partial v_{54}}{\partial x_{11}} \leqslant 0$	$\frac{\partial v_{54}}{\partial x_{30}} > 0$	$\frac{\partial v_{54}}{\partial x_{31}} < 0$		Phosphoenolpyruvate synthase (PpsA))	
$v_{55}(x_{30}, x_{31}, x_{32}, x_{34})$	$\frac{\partial v_{55}}{\partial x_{30}} > 0$	$\frac{\partial v_{55}}{\partial x_{31}} < 0$	$\frac{\partial v_{55}}{\partial x_{32}} > 0$	$\frac{\partial v_{55}}{\partial x_{34}} < 0$	Phosphotransferase system (PTS)	[4]
$v_{56}(x_{19})$	$\frac{\partial v_{56}}{\partial x_{19}} > 0$				RssB activation	[45, 47, 46]
$v_{57}(x_{12}, x_{34})$	$\frac{\partial v_{57}}{\partial x_{12}} > 0$	$\frac{\partial v_{57}}{\partial x_{34}} > 0$			cAMP synthesis	[56]
Complex formation reactions						
$v_{58}(x_{13}, x_{33}, x_{35})$	$\frac{\partial v_{58}}{\partial x_{13}} > 0$	$\frac{\partial v_{58}}{\partial x_{33}} > 0$	$\frac{\partial v_{58}}{\partial x_{35}} < 0$		Crp·cAMP complex formation	[55, 56]
$v_{59}(x_{21}, x_{24}, x_{36})$	$\frac{\partial v_{59}}{\partial x_{21}} > 0$	$\frac{\partial v_{59}}{\partial x_{24}} > 0$	$\frac{\partial v_{59}}{\partial x_{36}} < 0$		FruR·FBP complex formation	[21]
$v_{60}(x_{15}, x_{16}, x_{37})$	$\frac{\partial v_{60}}{\partial x_{15}} > 0$	$\frac{\partial v_{60}}{\partial x_{16}} > 0$	$\frac{\partial v_{60}}{\partial x_{37}} < 0$		GyrAB·GyrI complex formation	[57]
$v_{61}(x_{18}, x_{38}, x_{39})$	$\frac{\partial v_{61}}{\partial x_{18}} > 0$	$\frac{\partial v_{61}}{\partial x_{38}} > 0$	$\frac{\partial v_{61}}{\partial x_{39}} < 0$		RpoS·RssB* complex formation	[46]
Input/output fluxes						
$v_{62}(x_{33})$	$\frac{\partial v_{62}}{\partial x_{33}} > 0$				export cAMP	[55, 56]
$v_{63}(x_{31})$	$\frac{\partial v_{63}}{\partial x_{31}} > 0$				export Pyr	
$v_{64}(x_1, x_{22}, x_{23}, x_{34})$	$\frac{\partial v_{64}}{\partial x_1} < 0$	$\frac{\partial v_{64}}{\partial x_{22}} < 0$	$\frac{\partial v_{64}}{\partial x_{23}} > 0$	$\frac{\partial v_{64}}{\partial x_{34}} > 0$	Pentose-phosphate pathway	
$v_{65}(x_{34})$	$\frac{\partial v_{65}}{\partial x_{34}} > 0$				Glucose influx	[4]
v_{66}					Pyruvate influx	

Table 2: Table of reaction rates for the complete carbon assimilation model, with references to the literature for supporting evidence (continued). In the absence of evidence to the contrary, reaction rates are assumed to be reversible. In this case, the partial derivatives of rate laws with respect to enzyme concentrations can not be defined: they are positive in the glycolytic model and negative in the gluconeogenic model.

$$
\begin{aligned}
& \text{Glycolytic} - \text{gluconeogenic enzymes :} \\
\frac{dx_1}{dt} &= v_1 - v_{22}(x_1) \\
\frac{dx_2}{dt} &= v_2(x_{21}) - v_{23}(x_2) \\
\frac{dx_3}{dt} &= v_3 - v_{24}(x_3) \\
\frac{dx_4}{dt} &= v_4(x_{21}, x_{35}) - v_{25}(x_4) \\
\frac{dx_5}{dt} &= v_5 - v_{26}(x_5) \\
\frac{dx_6}{dt} &= v_6(x_{21}, x_{35}) - v_{27}(x_6) \\
\frac{dx_7}{dt} &= v_7(x_{21}, x_{35}) - v_{28}(x_7) \\
\frac{dx_8}{dt} &= v_8 - v_{29}(x_8) \\
\frac{dx_9}{dt} &= v_9(x_{21}) - v_{30}(x_9) \\
\frac{dx_{10}}{dt} &= v_{10}(x_{21}) - v_{31}(x_{10}) \\
\frac{dx_{11}}{dt} &= v_{11}(x_{21}) - v_{32}(x_{11}) \\
& \\
& \text{Global regulators :} \\
\frac{dx_{12}}{dt} &= v_{12}(x_{35}) - v_{33}(x_{12}) \\
\frac{dx_{13}}{dt} &= v_{13}(x_{14}, x_{35}) - v_{34}(x_{13}) - v_{58}(x_{13}, x_{33}, x_{35}) \\
\frac{dx_{14}}{dt} &= v_{14}(x_{14}, x_{35}, x_{40}) - v_{35}(x_{14}) \\
\frac{dx_{15}}{dt} &= v_{15}(x_{14}, x_{40}) - v_{36}(x_{15}) - v_{60}(x_{15}, x_{16}, x_{37}) \\
\frac{dx_{16}}{dt} &= v_{16}(x_{18}, x_{35}) - v_{37}(x_{16}) - v_{60}(x_{15}, x_{16}, x_{37}) \\
\frac{dx_{17}}{dt} &= v_{17}(x_{14}, x_{18}, x_{40}) - v_{38}(x_{17}) \\
\frac{dx_{18}}{dt} &= v_{18} - v_{39}(x_{18}) - v_{61}(x_{18}, x_{38}, x_{39}) \\
\frac{dx_{19}}{dt} &= v_{19}(x_{18}) - v_{41}(x_{19}) - v_{56}(x_{19}) \\
\frac{dx_{20}}{dt} &= v_{20}(x_{14}) - v_{42}(x_{20}) \\
\frac{dx_{21}}{dt} &= v_{21}(x_{21}) - v_{43}(x_{21}) - v_{59}(x_{21}, x_{24}, x_{36})
\end{aligned}
$$

Table 3: Model equations for the complete carbon assimilation model. The model consists of 40 variables and 66 reaction rates.

$$
\begin{aligned}
& \text{Metabolites :} \\
\frac{dx_{22}}{dt} &= v_{65}(x_{34}) - v_{44}(x_1, x_{22}, x_{23}) - v_{64}(x_1, x_{22}, x_{23}, x_{34}) \\
\frac{dx_{23}}{dt} &= v_{44}(x_1, x_{22}, x_{23}) + v_{46}(x_3, x_{22}, x_{24}, x_{30}) + \frac{4}{9}\, v_{64}(x_1, x_{22}, x_{23}, x_{34}) - v_{45}(x_2, x_{23}, x_{30}) \\
\frac{dx_{24}}{dt} &= v_{45}(x_2, x_{23}, x_{30}) - v_{47}(x_4, x_{24}, x_{25}) - v_{46}(x_3, x_{22}, x_{24}, x_{30}) - v_{59}(x_{21}, x_{24}, x_{36}) \\
\frac{dx_{25}}{dt} &= v_{47}(x_4, x_{24}, x_{25}) - v_{48}(x_5, x_{25}, x_{26}) \\
\frac{dx_{26}}{dt} &= v_{47}(x_4, x_{24}, x_{25}) + v_{48}(x_5, x_{25}, x_{26}) + \frac{2}{9}\, v_{64}(x_1, x_{22}, x_{23}, x_{34}) - v_{49}(x_6, x_{26}, x_{27}) \\
\frac{dx_{27}}{dt} &= v_{49}(x_6, x_{26}, x_{27}) - v_{50}(x_7, x_{27}, x_{28}) \\
\frac{dx_{28}}{dt} &= v_{50}(x_7, x_{27}, x_{28}) - v_{51}(x_8, x_{28}, x_{29}) \\
\frac{dx_{29}}{dt} &= v_{51}(x_8, x_{28}, x_{29}) - v_{52}(x_9, x_{29}, x_{30}) \\
\frac{dx_{30}}{dt} &= v_{52}(x_9, x_{29}, x_{30}) + v_{54}(x_{11}, x_{30}, x_{31}) - v_{53}(x_{10}, x_{30}, x_{24}) \\
\frac{dx_{31}}{dt} &= v_{66} + v_{53}(x_{10}, x_{30}, x_{24}) - v_{54}(x_{11}, x_{30}, x_{31}) - v_{63}(x_{31}) + v_{55}(x_{30}, x_{31}, x_{32}, x_{34}) \\
\frac{dx_{32}}{dt} &= v_{65}(x_{34}) - v_{55}(x_{30}, x_{31}, x_{32}, x_{34}) \\
\frac{dx_{33}}{dt} &= v_{57}(x_{12}, x_{34}) - v_{62}(x_{33}) - v_{58}(x_{13}, x_{33}, x_{35}) \\
& \\
& \text{Protein complexes and DNA supercoiling :} \\
\frac{dx_{34}}{dt} &= v_{55}(x_{30}, x_{31}, x_{32}, x_{34}) - v_{65}(x_{34}) \\
\frac{dx_{35}}{dt} &= v_{58}(x_{13}, x_{33}, x_{35}) - v_{34}(x_{35}) \\
\frac{dx_{36}}{dt} &= v_{59}(x_{21}, x_{24}, x_{36}) - v_{43}(x_{36}) \\
\frac{dx_{37}}{dt} &= v_{60}(x_{15}, x_{16}, x_{37}) - v_{36}(x_{37}) \\
\frac{dx_{38}}{dt} &= v_{56}(x_{19}) - v_{61}(x_{18}, x_{38}, x_{39}) + v_{40}(x_{39}) - v_{41}(x_{38}) \\
\frac{dx_{39}}{dt} &= v_{61}(x_{18}, x_{38}, x_{39}) - v_{40}(x_{39}) - v_{39}(x_{39}) \\
x_{40} &= a + b\, \frac{x_{15}}{x_{17}}
\end{aligned}
$$

Table 3: Model equations for the complete carbon assimilation model (continued).

Name	Variable	Definition
	Slow variables	
Pgi	x_1^s	x_1
PfkA	x_2^s	x_2
Fbp	x_3^s	x_3
FbaA	x_4^s	x_4
TpiA	x_5^s	x_5
GapA	x_6^s	x_6
Pgk	x_7^s	x_7
GpmI	x_8^s	x_8
Eno	x_9^s	x_9
PykF	x_{10}^s	x_{10}
PpsA	x_{11}^s	x_{11}
Cya	x_{12}^s	x_{12}
Crp	x_{13}^s	$x_{13} + x_{35}$
Fis	x_{14}^s	x_{14}
GyrAB	x_{15}^s	$x_{15} + x_{37}$
GyrI	x_{16}^s	$x_{16} + x_{37}$
TopA	x_{17}^s	x_{17}
RpoS	x_{18}^s	$x_{18} + x_{38} + x_{39}$
RssB	x_{19}^s	$x_{19} + x_{39}$
Stable RNAs	x_{20}^s	x_{20}
FruR	x_{21}^s	$x_{21} + x_{36}$
	Fast variables	
G6P	x_1^f	x_{22}
F6P	x_2^f	x_{23}
FBP	x_3^f	x_{24}
DHAP/ G3P	x_4^f	$x_{25} + x_{26}$
DPG	x_5^f	x_{27}
3PG	x_6^f	x_{28}
2PG	x_7^f	x_{29}
PEP	x_8^f	x_{30}
Pyr	x_9^f	x_{31}
PTSp	x_{10}^f	x_{34}
cAMP	x_{11}^f	x_{33}
Crp·cAMP	x_{12}^f	x_{35}
FruR·FBP	x_{13}^f	x_{36}
GyrAB·GyrI	x_{14}^f	x_{37}
RssB*·free	x_{15}^f	x_{38}
RpoS·RssB*	x_{16}^f	x_{39}
DNA supercoiling	x_{17}^f	x_{40}

Table 4: Table of fast and slow variables for the reformulated carbon assimilation model. The definition of the new fast and slow variables in terms of the original ones is shown.

Slow system

$$
\begin{aligned}
\frac{dx_1^s}{dt} &= v_1 - v_{22}(x_1^s) = 0\\
\frac{dx_2^s}{dt} &= v_2(x_{21}^s, x_{13}^f) - v_{23}(x_2^s)\\
\frac{dx_3^s}{dt} &= v_3 - v_{24}(x_3^s) = 0\\
\frac{dx_4^s}{dt} &= v_4(x_{21}^s, x_{13}^f, x_{12}^f) - v_{25}(x_4^s)\\
\frac{dx_5^s}{dt} &= v_5 - v_{26}(x_5^s) = 0\\
\frac{dx_6^s}{dt} &= v_6(x_{21}^s, x_{13}^f, x_{12}^f) - v_{27}(x_6^s)\\
\frac{dx_7^s}{dt} &= v_7(x_{21}^s, x_{13}^f, x_{12}^f) - v_{28}(x_7^s)\\
\frac{dx_8^s}{dt} &= v_8 - v_{29}(x_8^s) = 0\\
\frac{dx_9^s}{dt} &= v_9(x_{21}^s, x_{13}^f) - v_{30}(x_9^s)\\
\frac{dx_{10}^s}{dt} &= v_{10}(x_{21}^s, x_{13}^f) - v_{31}(x_{10}^s)\\
\frac{dx_{11}^s}{dt} &= v_{11}(x_{21}^s, x_{13}^f) - v_{32}(x_{11}^s)\\
\frac{dx_{12}^s}{dt} &= v_{12}(x_{12}^f) - v_{33}(x_{12}^s)\\
\frac{dx_{13}^s}{dt} &= v_{13}(x_{14}^s, x_{12}^f) - v_{34}(x_{13}^s)\\
\frac{dx_{14}^s}{dt} &= v_{14}(x_{14}^s, x_{12}^f, x_{17}^f) - v_{35}(x_{14}^s)\\
\frac{dx_{15}^s}{dt} &= v_{15}(x_{14}^s, x_{17}^f) - v_{36}(x_{15}^s)\\
\frac{dx_{16}^s}{dt} &= v_{16}(x_{18}^s, x_{12}^f) - v_{37}(x_{16}^s)\\
\frac{dx_{17}^s}{dt} &= v_{17}(x_{14}^s, x_{18}^s, x_{17}^f) - v_{38}(x_{17}^s)\\
\frac{dx_{18}^s}{dt} &= v_{18} - v_{39}(x_{18}^s) - v_{40}(x_{16}^f)\\
\frac{dx_{19}^s}{dt} &= v_{19}(x_{18}^s) - v_{41}(x_{19}^s) - v_{39}(x_{16}^f)\\
\frac{dx_{20}^s}{dt} &= v_{20}(x_{14}^s) - v_{42}(x_{20}^s)\\
\frac{dx_{21}^s}{dt} &= v_{21}(x_{21}^s, x_{13}^f) - v_{43}(x_{21}^s)
\end{aligned}
$$

Table 5: Reduced carbon assimilation model with slow and fast subsystem. The concentrations of constitutive proteins, *i.e.*, those of which the genes are not regulated in the network, are assumed constant and thus fixed at the steady-state value. This is notably the case for variables x_1^s, x_3^s, x_5^s, and x_8^s. Under the QSS approximation, the fast variables are fixed at their quasi-steady-state value.

Fast system

$$\frac{dx_1^f}{dt} = v_{65}(x_{10}^f) - v_{44}(x_1^s, x_1^f, x_2^f) - v_{64}(x_1^s, x_1^f, x_2^f, x_{10}^f) = 0$$

$$\frac{dx_2^f}{dt} = v_{44}(x_1^s, x_1^f, x_2^f) + v_{46}(x_3^s, x_1^f, x_3^f, x_8^f) + \frac{4}{9}\, v_{64}(x_1^s, x_1^f, x_2^f, x_{10}^f) - v_{45}(x_2^s, x_2^f, x_8^f) = 0$$

$$\frac{dx_3^f}{dt} = v_{45}(x_2^s, x_2^f, x_8^f) - v_{47}(x_4^s, x_3^f, x_4^f) - v_{46}(x_3^s, x_1^f, x_3^f, x_8^f) - v_{59}(x_{21}^s, x_3^f, x_{13}^f) = 0$$

$$\frac{dx_4^f}{dt} = 2\, v_{47}(x_4^s, x_3^f, x_4^f) + \frac{2}{9}\, v_{64}(x_1^s, x_1^f, x_2^f, x_{10}^f) - v_{49}(x_6^s, x_4^f, x_5^f) = 0$$

$$\frac{dx_5^f}{dt} = v_{49}(x_6^s, x_4^f, x_5^f) - v_{50}(x_7^s, x_5^f, x_6^f) = 0$$

$$\frac{dx_6^f}{dt} = v_{50}(x_7^s, x_5^f, x_6^f) - v_{51}(x_8^s, x_6^f, x_7^f) = 0$$

$$\frac{dx_7^f}{dt} = v_{51}(x_8^s, x_6^f, x_7^f) - v_{52}(x_9^s, x_7^f, x_8^f) = 0$$

$$\frac{dx_8^f}{dt} = v_{52}(x_9^s, x_7^f, x_8^f) + v_{54}(x_{11}^s, x_8^f, x_9^f) - v_{53}(x_{10}^s, x_8^f, x_3^f) = 0$$

$$\frac{dx_9^f}{dt} = v_{66} + v_{53}(x_{10}^s, x_8^f, x_3^f) - v_{54}(x_{11}^s, x_8^f, x_9^f) - v_{63}(x_9^f) + v_{55}(x_8^f, x_9^f, x_{10}^f) = 0$$

$$\frac{dx_{10}^f}{dt} = v_{55}(x_8^f, x_9^f, x_{10}^f) - v_{65}(x_{10}^f) = 0$$

$$\frac{dx_{11}^f}{dt} = v_{57}(x_{12}^s, x_{10}^f) - v_{62}(x_{11}^f) - v_{58}(x_{13}^s, x_{11}^f, x_{12}^f) = 0$$

$$\frac{dx_{12}^f}{dt} = v_{58}(x_{13}^s, x_{11}^f, x_{12}^f) = 0$$

$$\frac{dx_{13}^f}{dt} = v_{59}(x_{21}^s, x_3^f, x_{13}^f) = 0$$

$$\frac{dx_{14}^f}{dt} = v_{60}(x_{15}^s, x_{16}^s, x_{14}^f) = 0$$

$$\frac{dx_{15}^f}{dt} = v_{56}(x_{19}^s) - v_{61}(x_{18}^s, x_{15}^f, x_{16}^f) + v_{40}(x_{16}^f) = 0$$

$$\frac{dx_{16}^f}{dt} = v_{61}(x_{18}^s, x_{15}^f, x_{16}^f) - v_{40}(x_{16}^f) = 0$$

$$x_{17}^f = a + b\, \frac{x_{15}^s}{x_{17}^s}$$

Table 5: Reduced carbon assimilation model with slow and fast subsystem (continued).

		Regulators														
		FbaA	GapA	Pgk	Eno	PpsA	Cya	Crp	Fis	GyrAB	GyrI	TopA	RpoS	RssB	Stable RNAs	FruR
Genes	*fbaA*	0 (+)	0 (+)	0 (+)	0 (+)	+	+	+	0	0	0	0	0	0	0	-
	gapA	0 (+)	0 (+)	0 (+)	0 (+)	+	+	+	0	0	0	0	0	0	0	-
	pgk	0 (+)	0 (+)	0 (+)	0 (+)	+	+	+	0	0	0	0	0	0	0	-
	eno	0 (+)	0 (+)	0 (+)	0 (+)	0	0	0	0	0	0	0	0	0	0	-
	ppsA	0 (-)	0 (-)	0 (-)	0 (-)	0	0	0	0	0	0	0	0	0	0	+
	cya	0	0	0	0	-	-	-	0	0	0	0	0	0	0	0
	crp	0	0	0	0	+	+	+	-	0	0	0	0	0	0	0
	fis	0	0	0	0	0	-	-	-	+	-	-	0	0	0	0
	gyrAB	0	0	0	0	0	0	0	-	-	+	+	0	0	0	0
	gyrI	0	0	0	0	0	+	+	0	0	0	0	+	0	0	0
	topA	0	0	0	0	0	0	0	+	+	-	-	+	0	0	0
	rpoS	0	0	0	0	0	0	0	0	0	0	0	0	-	0	0
	rssB	0	0	0	0	0	0	0	0	0	0	0	+	0	0	0
	rrn	0	0	0	0	0	0	0	+	0	0	0	0	0	0	0
	fruR	0 (+)	0 (+)	0 (+)	0 (+)	0	0	0	0	0	0	0	0	0	0	-

Table 6: Interaction matrix of the gene regulatory network for the gluconeogenic mode. Plus signs stand for activation of a gene by a regulator, and minus signs for inhibition. Signs in brackets correspond to interactions whose signs are different in the case of allosteric regulation (that is, they appear when using model $\mathcal{M}^2_{neo}$, instead of model $\mathcal{M}^1_{neo}$).
